# Meta-analysis on comparative impact of behavioral, information, and monetary interventions on energy-efficient appliance adoption

**DOI:** 10.1093/pnasnexus/pgag129

**Published:** 2026-05-20

**Authors:** Tarun M Khanna, Diana Danilenko, Lukas Tomberg, Sven Hansteen, Mark A Andor, Paul Marvin Lohmann, Leo Ruesche Neggia, Jan C Minx

**Affiliations:** School of Public Policy and Global Affairs, The University of British Columbia, 6476 NW Marine Dr, Vancouver, BC V6T 1Z2, Canada; Potsdam Institute for Climate Impact Research, Berlin 10829, Germany; RWI—Leibniz Institute for Economic Research, Essen 45128, Germany; RWI—Leibniz Institute for Economic Research, Essen 45128, Germany; RWI—Leibniz Institute for Economic Research, Essen 45128, Germany; Ruhr University Bochum, Bochum 44801, Germany; El-Erian Institute of Behavioural Economics and Policy, University of Cambridge, Trumpington Street, Cambridge CB2 1AG, United Kingdom; Sciences Po, Paris 75007, France; Potsdam Institute for Climate Impact Research, Berlin 10829, Germany

## Abstract

Achieving energy efficiency in households is essential for climate mitigation, yet adoption remains low despite the availability of cost-effective technologies. This systematic review and meta-analysis synthesizes evidence from experimental and quasi-experimental studies assessing the efficacy of behavioral, information, and monetary interventions in efficient appliance adoption across three outcomes: purchase decisions, market share, and postpurchase energy consumption. The interventions yield small to medium average effects for purchase decisions, with labeling and information strategies performing on par with monetary interventions; loans show no discernible impact. Results are statistically robust but may be overstated due to reliance on stated preferences and study biases. Importantly, market-level effects and realized energy savings remain understudied, but the limited evidence suggests the presence of rebound effects in energy savings depending on the context. These findings provide program implementers with synthesized evidence on intervention effectiveness that should be weighed against implementation cost and scalability. Future research should cover the effects of interventions on economic welfare. Greater transparency in costs and welfare effects is essential to inform policy design and improve the marginal value of public expenditure in energy efficiency.

Significance statement Household energy efficiency is vital for climate action, yet voluntary uptake remains insufficient. This study aggregates experimental and quasi-experimental evidence to assess the effectiveness of behavioral, information, and monetary interventions in efficient appliance adoption. We find that information interventions like labeling can match the impact of monetary interventions, while loans are largely ineffective. However, evidence suggests rebound effects limit the extent of realized energy savings postpurchase. These insights guide policymakers toward scalable, cost-effective interventions for greater climate impact. Our findings advance understanding of energy efficiency policy design and identify priorities for future research and evaluation.

## Introduction

Sustainable Development Goal (SDG) 7.3 calls for doubling the global rate of improvement in energy efficiency compared with the 1990–2010 baseline. Improving energy efficiency is not only key to achieving SDG 7 and ensuring universal energy access but is also a critical component of mitigation pathways to keep global warming to below 2° (1–5). In fact, lower levels of energy consumption are a key feature of scenarios that tend to perform better in achieving other SDGs and limiting transitional risks (6, 7). But despite having the requisite technologies, the world is not on track to achieving the energy efficiency goals for 2030 (5).

Investments in energy efficiency are plagued by the so-called energy efficiency gap: households do not sufficiently invest in energy efficiency even though such investments promise substantial monetary benefits (see Gerarden et al. (8) for a review). The reasons underlying the energy efficiency gap are diverse, such as the long-time horizon until such investments pay off, risk aversion, hassle costs, information asymmetries, or the lack of adequate pricing of externalities. To address these, governments have adopted a diverse set of policies—monetary interventions (e.g. rebates), information strategies (e.g. labeling), and behavioral interventions (e.g. feedback)—to incentivize investments in energy efficiency.

Understanding which of these different policy interventions work and under what conditions is critical for climate and energy policy design as well as advancing research in the field (9). Evidence synthesis methods such as systematic reviews and meta-analyses can provide rigorous, transparent, timely, and fit-for-purpose evidence to decision-makers (10) while minimizing the biases common in traditional literature reviews (11). The lack of such comprehensive systematic review evidence on a multitude of policy questions hampers our ability to learn from implemented climate policies (9–12).

There have been few attempts to assess the existing evidence on the efficacy of different policy interventions relating to energy efficiency. Traditional literature reviews have described the economics of energy efficiency and estimated the size of the efficiency gap (13–15). A systematic review by Andor and Fels (16) qualitatively reviewed studies that evaluate the effect of information interventions (labels) on willingness to pay (WTP) for energy-efficient appliances, energy-saving potential, and hypothetical purchase decisions regarding energy-efficient appliances. Nisa et al. (17) conducted a meta-analysis of the impact of behavioral interventions on various energy-related outcomes but included only four studies on energy efficiency. Other systematic evidence syntheses have studied behaviors that motivate reduction in short-term energy use (18–23) but ignore the implications of interventions on decisions with long-term implications like energy efficiency investments (24, 25).

Here, we fill this gap through a machine-learning (MI)-enhanced systematic review and meta-analysis of the experimental and quasi-experimental literature that investigates the impact of monetary, information, and behavioral strategies on uptake and postpurchase use of energy-efficient appliances in households. Our contribution to the literature is 4-fold. First, no previous review has focused on household appliances, though they account for up to 55–65% of the residential electricity consumption in the European Union (EU) (26) and in the United States (27) and governments across the world have allocated around USD 264 billion since 2020 (28) toward promoting efficiency in households. Second, we bring together three strands of literature: studies that evaluate the impact of interventions on WTP for efficient appliances, studies that estimate their impact on overall sales share of efficient appliances, and studies that quantify the energy savings postpurchase of efficient appliances to provide complementary insights. Third, we use a mix of quantitative, mixed-Bayesian meta-analysis methods, and narrative review to compare the effectiveness of policies. Unlike traditional reviews, we are inclusive but use risk of bias and publication bias assessment to account for study quality. Fourth, all the information collected and code developed are publicly available to ensure transparency and reproducibility in line with Open Synthesis principles (29, 30).

We find that interventions targeting purchase decisions for energy-efficient appliances have a small-to-medium–sized average treatment effect, but an analysis of heterogeneity in effect sizes suggests that self-reported data and reporting biases exaggerate results. Behavior- and information-based strategies are as effective as monetary interventions in influencing purchase decisions, while loans are ineffective. The limited evidence on effect of interventions on overall sales suggests weak and heterogeneous effects. Lastly, the evidence on energy use postpurchase suggests that households reduce energy consumption when they purchase more efficient appliances but rebound effects limit total reductions. Overall, unlike prior reviews that have shown interventions to be successful in influencing short-term behaviors, the efficacy of interventions to influence energy efficiency adoption and energy consumption thereafter needs further examination.

### A diverse and clustered evidence base

**Figure 1:**
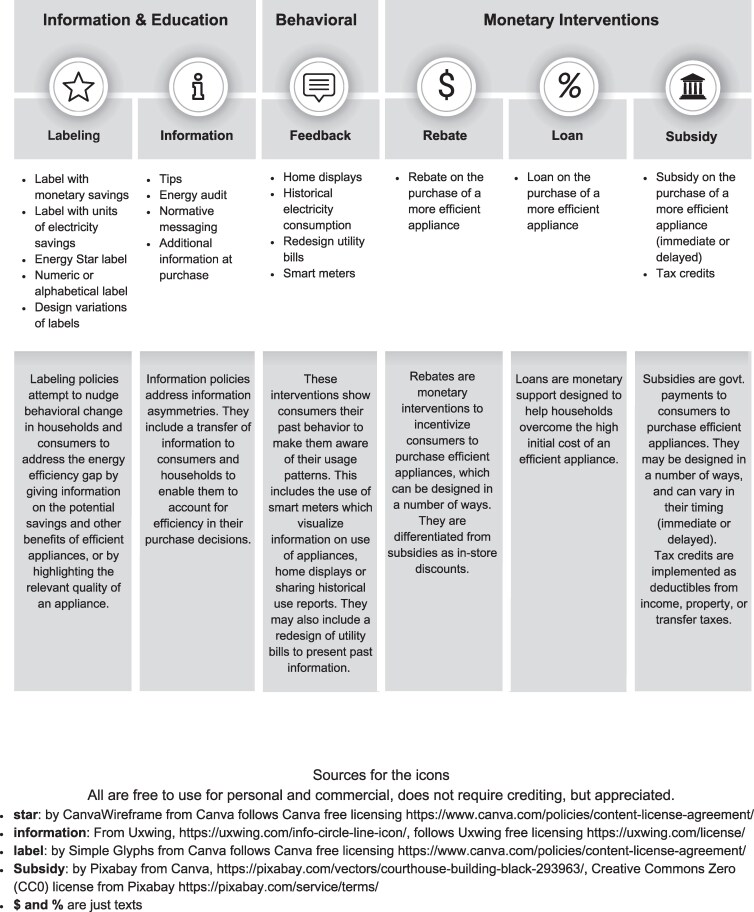
Classification scheme for interventions included in the analysis.

This review follows state-of-the-art systematic review guidelines and open-synthesis principles (29, 30) (see Materials and methods and the preregistered review protocol (31)) to answer the following review questions: are the interventions targeting the adoption of efficient appliances by households generally effective? Which category of interventions is most effective? How does energy consumption change after adoption of efficient appliances through such interventions? The interventions used in the studies were classified into information and education (information provision, label, and audits); behavioral interventions (choice architecture and feedback); and monetary interventions (rebates, subsidies, and loans) following previous studies in energy use among households (Fig. 1 (18–20)).

To address these questions, we identified studies that quantitatively assess the effects of behavioral, information, or monetary interventions on household purchases of energy-efficient appliances, including their impacts on the market share of efficient products. We also include studies that estimate subsequent reductions in household energy consumption attributable to the adoption of energy-efficient appliances when they are incentivized through policy interventions (see Table S2 for inclusion and exclusion criteria). We exclude studies in which interventions directly target household energy use—such as Home Energy Reports or time-of-use pricing—for three reasons: (i) although such interventions may indirectly influence appliance purchasing decisions, they do not isolate the contribution of energy-efficient appliances to changes in household energy consumption; (ii) they are not explicitly designed to promote energy-efficient appliance adoption and therefore do not allow identification of the effects of such intervention on energy efficiency investments; and (iii) their impacts have already been comprehensively synthesized in prior meta-analyses (17–20, 23). No previous reviews have focused specifically on appliance purchase and related energy savings, apart from Nisa et al. (17) which included four studies on this outcome variable (Table S3). We searched for relevant studies in the five major bibliographic databases (Web of Science, Scopus, JSTOR, RePec, and Google Scholar) returning over 30,000 unique records after deduplication. We then applied an ML-enabled prioritized screening approach (32, 33) to search for relevant experimental and quasi-experimental studies (see Materials and methods) that is able to find considerably more relevant evidence than conventional systematic as well as traditional reviews (18). Overall, we identified a total of 72 relevant studies after full-text screening and extracted 351 effect sizes from them, corresponding to an average of about five effects per study.

The identified studies differ in three important dimensions: the dependent variable studied, the intervention used, and the appliance considered. As shown in Table 1, 53 studies examine a household's or an individual's decision to purchase energy-efficient appliances, while only six estimate the aggregate effects on the market or sales share of efficient appliances. Fourteen studies examine household's energy consumption postpurchase following such interventions. The size of the evidence base informs our review approach: we conduct a quantitative meta-analysis of the studies on purchase decisions given the relatively large sample size and a narrative review of the small sample of market-share and energy-use studies. Evidence is also scattered unevenly across interventions. Though studies often use a combination of interventions, the most widely used intervention in the literature is labeling followed by other information strategies and monetary interventions. Refrigerators are the most frequently studied appliance in our sample, along with other high energy use appliances like water heaters and air conditioners. Clothes dryers, dishwashers, and televisions (TVs) noticeably remain underinvestigated, and only some evidence is available on newer appliances like heat pumps.

**Table 1:** Overview of the primary studies classified by the key dependent variable, appliance, and intervention.

	Probability of purchase, WTP	Market share/aggregate effects	Energy use
Total	53 studies (264)	6 studies (34)	14 studies (50)
Approach to synthesis	Meta-analysis	Descriptive review	Descriptive review
Interventions	Label (133) Information (53) Rebate (34) Subsidy (30) Loan (12) Defaults (2)	Rebate (15) Label (12) Subsidy (7)	Label (17) Rebate (20) Standards (7) Subsidy (4) Information (2)
Appliances	Air conditioner (37) Refrigerator (76) Water heater (43) Lighting (34) Heat pump (21) Clothes dryer (19) Washing machine (18) TV (12) Dishwasher (4)	Refrigerator (19) Washing machine (8) Dishwasher (4) Air conditioner (3)	Air conditioner (23) Refrigerator (23) Lighting (9) Heat pump (5) Washing machine (1) Lighting (9) Dishwasher (2) Refrigerator (4) TV (3) Water heater (3)

Figure in parentheses is the corresponding number of effect sizes. Note that total number of studies is 72 but appears as 73 in the table as one study (34) has information on both energy use and market share.

The summary statistics for the overall dataset and that included in the meta-analysis are presented in Tables S4 and S5, respectively. The sample of studies encompasses experiments and programs in 25 countries. The earliest study dates back to the early 1980s, but around half of the sample is from studies conducted after 2016. Studies are spread around the globe: about 26% of the effect sizes come from studies in continental Europe and the United Kingdom, 31% from Asia, 24% from North and Central America, and the remaining 17% from Africa, Oceania and cross-country studies, but the focus on studies published in English is an important limitation of this review. Included studies rely on various study designs, where 45% of the observations come from studies employing quasi-experimental designs, 29% from experimental designs, and 8% come from pre-post studies. Another important aspect of the study design is whether the results are based on revealed behavior of households and individuals, for example, the actual purchase of an appliance, or whether the study asks the participants to self-report actions that they took, or if the study only reports the stated preference of the participants. About 36% of the observations in our sample are based on stated preferences, 45% on revealed preferences and the remaining 19% on self-reported behavior. Reliance on stated preferences and self-reported behavior is a limitation of the literature that we investigate in our analysis.

## Results

### Impact of interventions on purchase decisions

#### Quantitative synthesis of effect sizes

Fifty-three primary studies in our sample investigate the probability of purchasing, or WTP for, an efficient appliance over standard appliances with the exact specification varying across studies. We first standardized the effects by converting the estimates reported for each study to a standardized metric (Fisher's *Z*) and then used meta-analysis models to calculate the average effect across studies and the causes for heterogeneity in results across studies. We apply a multilevel random-effects model that allows us to deal with dependent effect sizes within and between studies flexibly (see Materials and methods).

**Figure 2:**
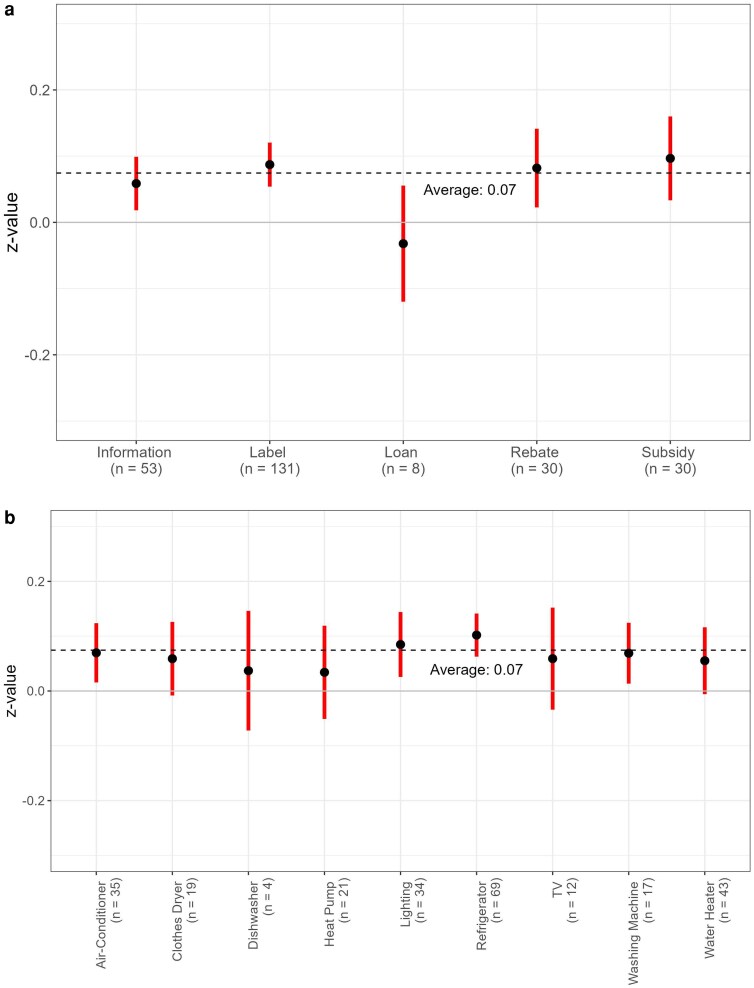
Average effect size along with the 95% CI across (a) interventions and (b) appliances. *Z* > 0 implies an increase in WTP or probability of purchase. The results are from a multilevel meta-regression model that accounts for publication bias and influential observations.

We find an overall small to medium average treatment effect of 0.07 (95% CI = 0.05–0.10) across interventions. The average effect sizes for information-based interventions (0.06, 95% CI = 0.02–0.10 for information and 0.09, 95% CI = 0.05–0.12 for labeling) are broadly comparable to monetary interventions (0.08, 95% CI = 0.02–0.14 for rebates and 0.10, 95% CI = 0.03–0.16 for subsidies) with overlapping CIs (Fig. 2). Loans are the only intervention category for which the average effect size is negative and not statistically significant. Only three studies provide estimates for this intervention and all report no significant effect of loans on stimulating purchase of efficient appliances (35, 36, 37). The CIs are in general larger for rebates and subsidies, due to the lower number of effect sizes compared with information-based interventions and a greater variation in results possibly reflecting the size of the incentive and the context in which it was offered. These average treatment effects were estimated after controlling for small-study/publication bias and potential outliers (see Materials and methods and Supplementary Note S3). Not adjusting for publication bias and outliers does not alter the results in a substantive manner, but the average effect sizes are larger for all categories of interventions and the average effects for rebates and subsidies have larger CIs (Tables S6 and S12).

Previous meta-analyses on the impact of interventions on household behaviors have reported similar average effects but higher effects for monetary interventions when compared with behavioral and information strategies (18, 19) but we do not find any statistical or substantive differences between these types of interventions here. We are not able to reject the null hypothesis that the effects are equivalent with a Wald test. On the one hand, it could be that this difference is attributable to the different contexts of these reviews. For example, in the context of energy consumption, specific price-based approaches such as time-of-use and critical peak pricing can be used, which could potentially be more effective than the rewards, rebates, and other monetary interventions that have been studied in the context of appliance purchase decisions.

Among the appliance categories, refrigerators, lighting, air conditioners, and washing machines have the strongest treatment effects that are also statistically significant (Fig. 2). The average effect size for other high electricity consumption appliances, namely water heaters and heat pumps are weak and not statistically significant. The differences across appliance categories, irrespective of energy intensity or cost of the appliance, are however not substantive and not statistically significant.

#### Heterogeneity

There is considerable unexplained heterogeneity in effect sizes that we extract from primary studies (*I*^2^ = 97–98% for random-effects model with intervention/appliance as a moderator variable). Estimates of the effect size may vary because of (i) variables that affect the WTP for appliances, e.g. type of intervention and appliance, location of the experiment, type of participant (homeowners vs. tenants), etc. or (ii) variables that represent the design elements or biases contained in primary studies (e.g. setting of the experiment, statistical techniques used for analysis, reporting biases, etc.) and included those also in the meta-analysis. We employed the state-of-the-art hybrid-frequentist Bayesian approach to identify the most important variables that could explain the heterogeneity in results out of a set of 16 potentially relevant variables and their 216 possible combinations. We employ variable selection based on Bayesian model averaging (BMA; specifically, the variables with posterior inclusion probability [PIP] >50%; see Materials and methods) and then use these identified variables in a frequentist model with clustered SEs to estimate the effect of variables on variation in effect sizes (results in Table 2).

**Table 2:** Results from frequentist meta-regression based on moderator variables identified through Bayesian model averaging.

Variable	Beta coefficient	SE	*P*-value
Participant type (base: Unspecified)			
Homeowners	−0.03	0.02	0.23
Residents	−0.11***	0.03	0.00
Intervention type (base: Information)			
Label	0.03	0.02	0.26
Loan	−0.11***	0.04	0.01
Rebate	0.08	0.05	0.08
Subsidy	0.03	0.04	0.38
Appliance (base: Refrigerator)			
Clothes dryer	−0.03	0.02	0.27
Dishwasher	−0.06	0.03	0.08
Heat pump	−0.15**	0.06	0.03
Lighting	0.02	0.03	0.51
Air conditioner	−0.08**	0.03	0.02
TV	−0.20***	0.05	0.00
Washing machine	−0.04	0.04	0.34
Water heater	−0.06	0.03	0.07
Incentive compatibility (base: Actual Behavior)			
Self-reported behavior	0.06	0.03	0.08
Stated preferences	0.13***	0.03	0.00
Reporting bias (base: Probably No)			
Probably Yes	0.09***	0.03	0.00
Out of sample bias (base: Probably No)			
Probably Yes	−0.01	0.02	0.78
Square of SE of *Z*	3.17***	0.42	0.00

The dependent variable is the standardized Fisher's *Z* calculated for each effect size reported by studies. *Z* > 0 indicates a higher WTP for energy-efficient appliances. The model estimated is ordinary least squares with clustered SEs (see Materials and methods for details). ***P ≤ 0.01; **P ≤ 0.05.

The heterogeneity analysis confirms that there is no statistical difference between the average effects of interventions except “Loans” that have lower effects (Table 2). There is some variation in the impact of these interventions by the appliance type: studies involving air conditioners and TV report smaller effects compared with refrigerators, the appliance with the largest share of effect sizes. We also find that residents, who are not homeowners, are less likely to purchase energy-efficient appliances compared with a group of unspecified individuals. None of the other variables that could drive heterogeneity in results, including location, energy prices, demographics of the household, environmental attitudes, or the year in which the study was conducted, were statistically significant.

We find that some elements of study design are statistically significant in explaining differences in outcomes reported. Studies that measured the impact of the interventions in terms of changes in self-reported behaviors or stated preferences of the subjects of the study reported higher impact when compared with studies that measured the change in actual purchase behavior in response to the interventions. The differences resulting from studies that employ the different incentive-compatibility designs are statistically significant (Table 2). This result is expected for two primary reasons. First, customers might tend to say that they would purchase a high-efficiency appliance more readily than they actually do. Second, any time or effort that the customer must expend to receive a rebate is not reflected in the stated preference data (35). The main benefit of using the stated preferences approach is full and complete control over the experimental setting; however, the choices are based on hypothetical situations, decisions are often made removed from any context and respondents are not financially committed to their choices, which may bias results upward (38).

#### Critical appraisal of primary studies

We critically appraised the evidence through: (i) qualitative assessment of studies for reporting and out of sample biases and (ii) evaluation of publication/small study bias and its relationship with heterogeneity in treatment effects. We critically assessed and recorded the potential out-of-sample and reporting biases in the studies during the coding process. Out-of-sample bias reflects confounding and external validity concerns, while reporting bias indicates selective reporting or errors. Approximately 29 and 12% of the effect sizes showed potential out-of-sample bias and higher risk of reporting bias respectively in the dataset on studies involving purchase probability and WTP. The BMA analysis and the meta-regression results (Table 2) showed that studies with reporting bias tended to report higher WTP, whereas out-of-sample bias showed no significant effect. We also assessed potential publication bias in the dataset, using funnel plots and Egger's regression (see Supplementary Note S2 and Fig. S4). We confirm the presence of potential publication bias, with more studies than expected reporting positive effects though correcting for it does not change the results materially. Nevertheless, all estimates in Fig. 2 and Table 2 are corrected for potential publication bias.

### Impact of interventions on market share of efficient appliances

Beyond the literature on purchases probability and WTP, six studies also investigate the impact of information and monetary interventions from different energy conservation programs on the overall market share of efficient appliances in the United States and Europe using econometric analysis (Fig. S3 presents forest plot of the extracted effect sizes). These studies provide some insight into the impact of real-world policies that scale-up interventions in an effort to promote energy efficiency. Two of these studies focus on rebates by federal and local governments in the United States for efficient appliances under ENERGY STAR, a voluntary labeling program. Datta and Filippini (39) estimate that the rebates increase the sales share of ENERGY STAR appliances between 9 and 18% based on average market share. The results are higher than the findings in Datta and Gulati (40) who find that, on average, rebates increase the sales of ENERGY STAR washing machines by 6% but do not have statistically significant impact on sales share of dishwashers and refrigerators. Datta and Filippini account for the endogeneity of the rebate policy itself, so the results may be considered more robust than Datta and Gulati. Houde and Aldy (34) evaluate rebates under another program in the United States—the State Energy Efficient Appliance Rebate Program, and find that rebate programs increased efficient appliance sales by 7–10% during the rebate period, which translates into a market share increase of energy star appliances of 1–2 percentage points. But they find that about 70% of the consumers who claimed the rebate would have bought the efficient appliance anyway, so rebates did not have a strong impact on attracting new consumers. An additional 15–20% of consumers changed the timing of their purchase of an efficient appliance by a few weeks. In contrast to these findings, Buettner and Madzharova (41) find that in the European context, rebates for energy efficient labeled refrigerators and washing machines increased the sales of the efficient appliances (though significant adverse effects on the sales of nonsubsidized products) and did more than just shift the timing of purchase. They find that rebate programs had a lasting positive impact on the energy efficiency of the stock of household appliances and argue this is because the European rebates were solely aimed at improving energy efficiency, whereas the American programs were aimed at stimulating consumer spending. Schleich et al. (42) investigate the effectiveness of minimum energy performance standards (MEPS) and energy labels in the EU. MEPS and energy labels are therefore considered pillars of the EU's strategy to achieve its energy efficiency and climate policy targets and studies that model energy demand pathways consider substantial reductions of 40–60% in energy demand of appliances (43). They find that MEPS and the energy labels together increased the sales share of cold appliances with an energy label of A+ and better by ∼15–38 percentage points, which is lower than the modeling estimates but higher than the impact of the labels themselves.

### Changes in energy consumption after purchase of efficient appliances

Increasing the sale of energy-efficient appliances is only a means to an end. Energy-efficient appliance adoption needs to translate into actual reductions in energy consumption to reduce energy related household carbon emissions. Theoretical and empirical literature (25, 44) has highlighted the possibility of so-called rebound effects, i.e. the phenomenon whereby efficiency gains lower the price per unit of energy services, leading to higher demand for those services and partially offsetting energy savings (e.g. using a more energy-efficient dryer more often). If the rebound effect is strong enough to increase rather than decrease energy consumption, it is termed as “backfire.” We identified 14 studies that evaluate the energy use of households after replacement of standard appliances with efficient appliances in response to subsidies or labeling interventions. The evidence base is diverse, with studies varying strongly in methodological rigor and reporting results from policies implemented in high-, middle-, and low-income countries (see Table S8 for full list).

Despite some differences in magnitude of the effect, all studies report some level of rebound effect. Savings in energy consumption are generally lower than engineering estimates that do not account for behavioral change, though most studies do not report “backfire” effects. “One set of studies” that compare the changes in energy consumption of households that owned an efficient vs. inefficient appliance in response to “information or labeling interventions” provide mixed results. Mizobuchi and Takeuchi (45) find that households that owned an efficient air conditioner in response to an information campaign reduced their consumption by ∼9% though the effect was statistically significant only for households that had recently purchased an air conditioner rather than replacing the existing air conditioner with a more efficient one. Liddle et al. (46) find 7.8% reduction in energy consumption after the purchase of a more efficient air conditioner inclusive of rebound effect. Sun (47), however, find that labeling schemes resulted in no statistical reduction in energy consumption in case of efficient air conditioners though there was reduction in use and energy consumption of efficient dishwashers. Cheng et al. (48) even finds that efficient labeled appliances (except refrigerators) are correlated with higher use and energy consumption when compared with unlabeled appliances in China, though their research design cannot establish causality. “A second set of studies” that investigate energy use postpurchase of an efficient appliance in response to a “monetary intervention” find a reduction in energy use but also significant rebound effects. Houde and Aldy (34) find that State Energy Efficient Appliance Rebate Program in the United States did not translate into any meaningful reduction in energy consumption as rebates led consumers to upgrade to less efficient appliances with more features. This is similar to findings of Yao et al. (49) who find that a subsidy for energy-efficient appliances in China led to an increase in the energy consumption of households though the study does not employ an experimental design that can isolate causality. Chuang et al. (50), however, find 4% reduction in overall energy use as a result of efficiency upgrades after accounting for rebound effects.

Literature indicates heterogeneity in the size of the rebound effect across appliances, incentive size, household, and country income levels. Chuang et al. (50), Cheng et al. (48), and Davis et al. (51) do not find any statistically significant or substantive reduction in energy consumption on upgrades to efficient air-conditioning, and Alberini et al. (52) do not find significant reductions for space heating. The authors argue that rebates allow households to upsize and defray the cost of bigger units, or of units that end up being used more. Hammerle and Burke (53) find a reduction in energy consumption for hot water heating when households are given subsidies to replace natural gas heaters with more efficient electric ones in Australia though they cannot establish causality. Houde and Aldy (34), Cheng et al. (48), and Chuang et al (50) find no reduction in consumption for dishwasher upgrades. Davis et al. (51), Houde and Aldy (34), Chuang et al. (50), and Naeher et al. (54), however, find reduction in energy consumption in response to subsidies for replacing old refrigerators. Naeher et al. (54), Chuang et al. (50), Chun and Jiang (55), and Shojaeddini and Gilbert (56) find reduction in energy consumption in response to subsidies for efficient lighting, though there is significant rebound effect. Alberini et al. (52) report that an increase in the size of the monetary interventions corresponds to a larger rebound effect. Shojaeddini and Gilbert (56) find that rebound effects are larger for low-income households and those in smaller homes. Davis et al. (51) investigate the effect of rebates for the adoption of efficient air conditioners in Mexico conclude that rebound effects are expected to be larger in developing countries, where the demand for energy services is suppressed by the ability to pay for these services.

## Discussion and conclusions

This systematic review and meta-analysis aggregates evidence from experimental and quasi-experimental studies that evaluate the impact of behavioral, information, and monetary interventions on the purchase of energy-efficient appliances, market share of efficient appliances, and savings in energy consumption postpurchase. By jointly discussing the effects of these interventions across different outcomes, we aim to provide complementary insights into their overall effectiveness.

Our meta-analysis finds that the studied interventions increase the purchase of energy-efficient appliances, showing a small to medium average effect size (average effect size measured as *Z*-score between 0.06 and 0.10). While these effects are statistically significant and robust to outliers and model selection, they may be overstated as about half of the effect sizes in our dataset come from studies that rely on stated preferences. Studies with higher risk of bias also tend to report larger effect sizes. Notably, the average effect sizes are smaller than average effect sizes reported by meta-analysis that study effect of similar interventions on other household behaviors (18, 57, 58). Additionally, contrary to findings from previous reviews (18, 59), monetary interventions do not outperform other strategies. In fact, among monetary interventions, loans for energy efficiency are found to be ineffective.

The relatively small average effect sizes in our meta-analysis underscore the need for a careful cost-effectiveness assessment before deploying an intervention. While our meta-analysis quantifies the effectiveness dimension, information on intervention costs is scarce. Literature suggests several contextual factors that influence cost-effectiveness: e.g. economies of scale may lower per-household costs when an intervention is rolled out across many households, potentially tipping the balance in favor of implementation even when effects are modest (60). Additionally, targeting frequently encountered decision points, such as energy-label displays on appliances or lighting, can magnify cumulative impact, as a small per-purchase effect is multiplied across many transactions (61). Program implementers should weigh locally available cost estimates against expected intervention effects. Beyond pure cost-effectiveness, interventions differ markedly in their welfare implications (62, 63). Changes in consumer surplus can be negative (e.g. higher upfront costs, reduced comfort, or social pressure) or positive when consumers value the information provided or appreciate assistance in cutting energy use (64, 65). Consequently, an intervention that fails conventional cost-effectiveness tests may still enhance overall welfare, and vice versa. To increase the informative value of meta-analyses for such questions, as also recommended in Andor and Fels (16), studies should be more transparent about the costs of interventions, scalability, and possibly welfare effects, so that these aspects can also be included in the quantitative research synthesis or the marginal value of public funds can then be estimated and compared (see, for example, Hahn et al. (66)). Evidence is also limited on key interventions such as loans or subsidies and despite the larger number of labeling and information-based measures deployed by governments, ex postpolicy evaluations of these programs that identify a causal effect are still scarce.

More research is also needed to fill the structural gap in primary evidence on the effects on market shares and energy consumption. The extant evidence suggests that the studied interventions have only a marginal impact on increasing the market share of efficient appliances, though at least one study indicates that combining these interventions with regulatory instruments could produce more substantial effects (42). While additional evidence is needed to confirm these findings, exploring how the studied interventions might be more effective as part of smart policy packages, e.g. around other regulatory instruments or standards, represents an important avenue to increase energy efficiency adoption. The scarce evidence on energy consumption suggests that rebound effects limit the effectiveness of such policies in driving reduction in energy consumption, which is consistent with the recent findings on energy efficiency improvements through home renovation (67). The heterogeneity in rebound effects is also expected to be larger in developing countries, for lower socioeconomic households, and for appliances where the demand for energy services is suppressed by the ability to pay for these services. In this sense, energy efficiency subsidies, even if they have a lower impact on energy consumption, can create welfare gains due to the increase in consumption of energy services, though the cost-effectiveness of such measures needs to be examined.

Closing these gaps in research could be fundamental for better understanding the role of behavioral, information, and monetary interventions in climate and energy policy.

## Materials and methods

### Literature search and data extraction

For data collection, we relied primarily on (i) string-based searches of bibliographic databases and (ii) a rolling review of existing literature reviews on the same topic. In compliance with recommendations for systematic evidence syntheses, we included several bibliographic databases in our search (Web of Science Core Collections Citation Indexes, Scopus, JSTOR, and RePEc), as well as the web-based academic search engine Google Scholar. The Web of Science Core Collection Citation Indexes included in our search were: Science Citation Index Expanded: 1900–present; Social Sciences Citation Index 1900–present; Arts and Humanities Citation Index: 1975–present; Conference Proceedings Citation Index-Science: 1990–present; Conference Proceedings Citation Index-Social Science and Humanities: 1990–present; and Emerging Sources Citation Index: 2015–present. The records pulled from JSTOR were limited to only publications after 1960 due to an inconsequential number of publications available from years before. We applied a comprehensive search string, which was developed based on the PICOS (population, intervention, comparator, outcome, and study design) approach recommended by the Campbell Collaboration (Table S1). The search string was developed reiteratively by checking the results of the search against a set of relevant studies previously identified. The search targeted studies examining household energy (or electricity) consumption and/or the purchase of energy-efficient appliances in the presence of one or more behavioral, information, or monetary interventions of interest (see Table S2 for inclusion–exclusion criteria). To make manual screening of relevant papers possible, an ML algorithm was applied that rated the studies based on the predicted relevance at the abstract level (Supplementary Note S3 and Fig. S2). After training the model with 32 previously known relevant study abstracts and a random sample of 500 study abstracts, several reiterations were performed, where a team of five reviewers screened the predicted most relevant abstracts manually. The abstracts were screened individually, however, to ensure consistency, two samples of 50 studies were screened by the five reviewers independently at the abstract level (Kappa = 0.68). We applied ML-assisted screening process with the implementation of statistical stopping criteria for screening these documents at an abstract level, which resulted in ∼85% work savings and a recall of 0.9 achieved at *P* = 0.018. In total, 5,861 studies were screened manually at the abstract level. Out of these, 241 studies were screened at the full-text level, and the final sample included 72 studies. The PRISMA flowchart for the screening and coding process is available in Fig. S1, as well as the complete list of studies included in the analysis (Table S9). Exclusion reasons for studies are given in the PRISMA flowchart. Four reviewers extracted the relevant data from the included studies following the guideline described in detail in a codebook (Table S11) and holding regular discussions of the coded fields to see what disagreements occurred, while making suitable adjustments to the codebook. A single reviewer double-checked the final data collected for all the included studies. The data was extracted manually using MS Excel.

### Standardizing effect sizes

The effect sizes for purchase probability and WTP reflected different dependent variables (e.g. choice of a high-efficiency class appliance, WTP for a high-efficiency class appliance, etc.), which were harmonized and analyzed together, whereas usage and market share effect sizes were studied separately. We harmonized the effect sizes following a standard procedure described by Ringquist (68) of first converting the recorded regression coefficients into correlation coefficients (*r*) using the total sample size, which we then converted to Fisher's *Z*. If a study applied the difference-of-means design, the standardized mean differences (or Cohen's *d*) were calculated first and then converted to Fisher's *Z*. The formulas for the described conversions are specified by Ringquist (68). These values were then used for meta-regressions. For the analysis on energy usage and market share impact, there were fewer studies available, and the dependent variables were homogeneous. Thus, the effect sizes on usage and market share were harmonized as percentage change estimates so that a descriptive analysis could be performed.

### Synthesis

After the harmonization of individual effect sizes through conversion to Fisher's *Z* (68), we implemented a random-effects model with intervention and appliance category as predictors. The “metafor” package in R was used to get the estimates from FE and REML model specifications. We present the estimates from a REML model in the main text, as the underlying effects in our sample are likely to be heterogenous, in which case a random-effects model is considered more appropriate than a fixed effects model (68, 69). Furthermore, as some of the studies in the sample reported multiple effect sizes were extracted from each study, we applied the multilevel random-effects model to account for possible interdependence of the effect sizes. We also checked that no single study or specific effect size disproportionately affected the model results. We calculated Cook's distance values for coefficients in the meta-regression. Observations with the highest values of this parameter were dropped (9 and 6 influential observations for intervention-specific and appliance-specific models, respectively). The results presented in the main text are after exclusion of the influential observations. Not excluding these observations does not materially change the results except that the CIs for the estimated effect sizes become larger (Table S6).

### Heterogeneity

There is considerable heterogeneity in effect sizes that we extract from primary studies (*I*^2^ = 97–98% for random-effects model with intervention/appliance as a moderator variable). Estimates of the effect size may vary both because of the variation in the underlying response to interventions or because of differences in characteristics of the studies, which we captured using 16 variables that we coded. The complete list and their summary statistics are presented in Tables S4 and S5. We use the following regression to estimate the relationship between the potential explanatory factors discussed above, and the WTP reported by the studies, standardized as *Z*-value, in our dataset:


$${\hat{Z}}_{ij}=\alpha +\;\overset{16}{\underset{k=1}{\sum }}\;\theta_{k}X_{k,ij}+\;\varepsilon_{ij},$$

where ${\hat{Z}}_{ij}$ is the *i*th estimate from the *j*th study, and $X_{k,ij}$ is the corresponding value of the *k*th explanatory variable. α is the intercept, θ is a vector of *k* parameters, and ε is a vector of error terms. The best model that contains all the relevant explanatory variables is selected using BMA that runs many regressions with different possible combinations of explanatory variables (70–72). We do not estimate all combinations of model specifications possible given our explanatory variables but rather employ the Markov chain Monte Carlo (specifically, the Metropolis–Hastings algorithm of the “bms” package for R by Zeugner and Feldkircher (73)), which walks through the most likely models. In the Bayesian setting, each coefficient is assigned a PIP that reflects the probability of the variable being included in the underlying model and is calculated as the sum of posterior model probabilities across all models in which the variable is included (74, 75). We employ the unit information g-prior that the regression coefficient is zero the same weight as one observation of the data (74) as the base prior, supplemented by the dilution prior that adjusts model probabilities by multiplying them by the determinant of the correlation matrix of the variables included in the model (71, 72). The results from the BMA are provided in Table S7. The tables show the PIP that indicates the relevance of each variable. Commonly, variables with a PIP >0.5 are interpreted to be relevant explanatory factors. To provide more readily interpretable results we run a hybrid-frequentist Bayesian model. We employ variable selection based on BMA, i.e. we only include the variables with PIP >50% and estimate the resulting model using an ordinary least squares model with clustered SEs. The results for this exercise are presented in Table 2.

## Supplementary Material

pgag129_Supplementary_Data

## Data Availability

The authors declare that the data supporting the findings of this study are available within the paper and its Supplementary material and is made available on GitHub (https://github.com/di-danilenko/appliances_interventions/tree/main) along with the code developed for the paper.

## Preprints

This manuscript was posted as a preprint at https://hdl.handle.net/10419/315481.2.
