# Supplementary material for: Meta-analysis on comparative impact of behavioral, information, and monetary interventions on energy-efficient appliance adoption
Source: PNAS Nexus. 2026 May 20;5(5):pgag129. doi: 10.1093/pnasnexus/pgag129 (PMC13189399; doi:10.1093/pnasnexus/pgag129)
Supplement: pgag129_Supplementary_Data [file pgag129_supplementary_data.pdf]

Supplementary Figure 1: PRISMA flowchart for search and screening of studies 2
Supplementary Figure 2: Graphical depiction of the results from machine learning prioritized screening and stopping criteria used for searching for the relevant literature... 3
Supplementary Figure 3: Forest plot showing average effect size with 95% confidence intervals across primary studies.. 4
Supplementary Figure 4: Funnel plots with standard error of each effect size on the y-axis and z-value of the effect size on the x-axis. 31

Supplementary Table 1: Search string applied to databases. Syntax is given for Web of Science Core Collection. 5
Supplementary Table 2: Inclusion/Exclusion criteria used for classifying studies................................... 6
Supplementary Table 3: Overview of previously published related literature.. 7
Supplementary Table 4: Detailed descriptive statistics for all coded effect sizes................................. 10
Supplementary Table 5: Detailed descriptive statistics for effect sizes on purchase probability (included in the meta-analysis).. 12
Supplementary Table 6: Results from meta-regression on purchase probability................................. 14
Supplementary Table 7: Results from the heterogeneity analysis using Bayesian model averaging..... 15
Supplementary Table 8: Studies that measure energy consumption post treatment.......................... 16
Supplementary Table 9: Complete list of studies included in the analysis 19
Supplementary Table 10: Average effect size by each study design employed................................... 23
Supplementary Table 11: Codebook used to record necessary information from the included studies 24
Supplementary Table 12: Average effect size across all interventions before and after correcting for publication bias... 31

Supplementary Notes 30
Supplementary References.. 33
References to included papers 34

Supplementary Figure 1: PRISMA flowchart for search and screening of studies

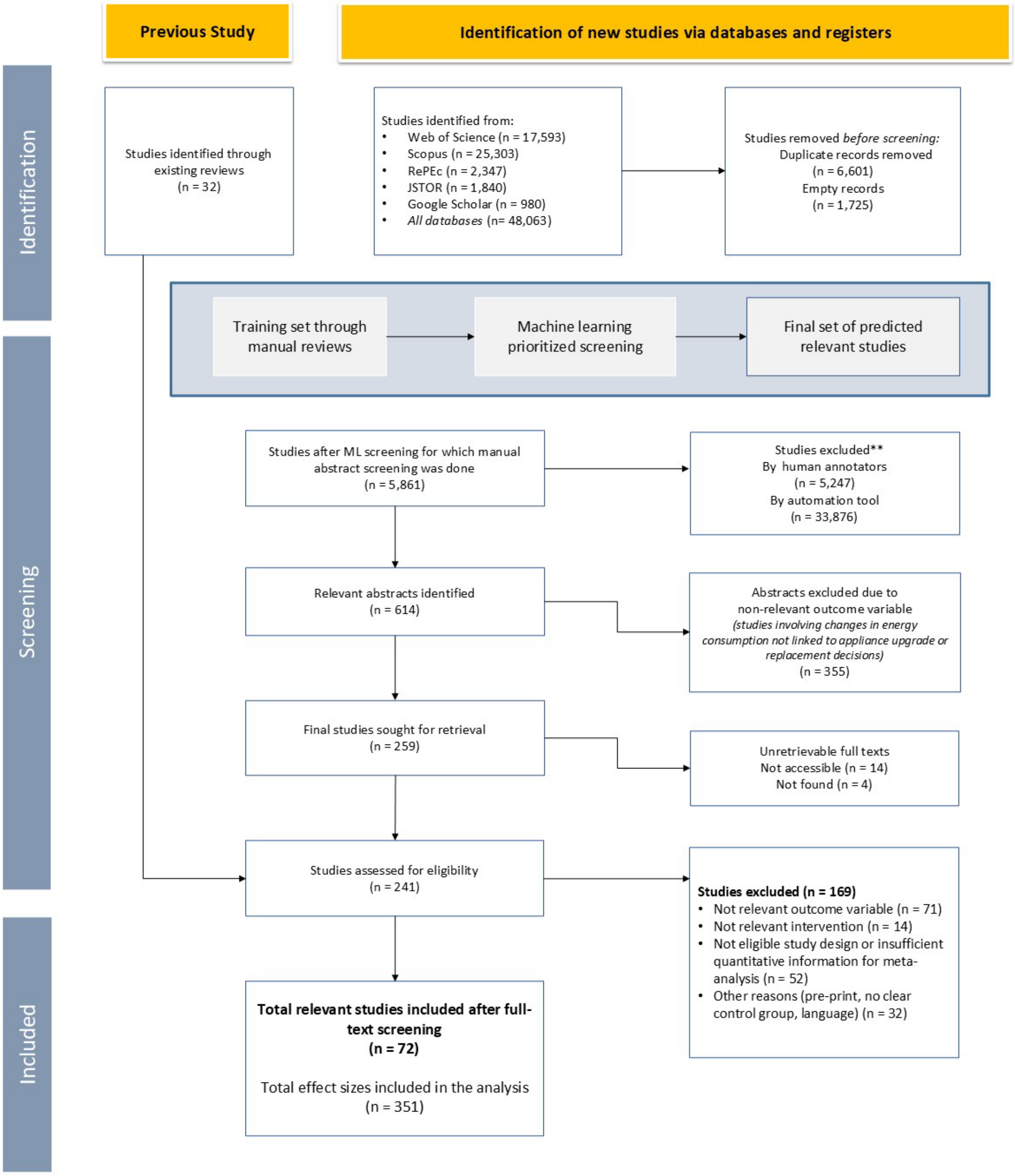

Exclusion reasons: studies that involved changes in energy consumption but not linked to appliance upgrades or replacement decisions (N= 355); studies that could not be retrieved at full text due to either access restrictions (N = 14) or general unavailability of full text due to incomplete records (N = 4); dependent variable outside of the scope of this study (N= 54, these studies mostly focused on building retrofits, or appliances that were not considered in this review, or dependent variables that were not in any way related to direct purchase decisions / observed energy usage); independent variable something other than an intervention of interest (N = 14, a common thread among these studies was to analyze the effect of socio-demographic characteristics on appliance purchase decisions which lies outside the scope of this analysis); irrelevant study design or incomplete uncertainty information (N = 52, most of these studies applied modelling as their main methodology; there were 10 theoretical papers, 3 literature reviews, and 3 case studies; 11 of these studies provided no quantitative estimates, and in 8 of them no uncertainty measurements could be calculated for the effect sizes); and language of the text was not English (N = 1).

Supplementary Figure 2: Graphical depiction of the results from machine learning prioritized screening and stopping criteria used for searching for the relevant literature

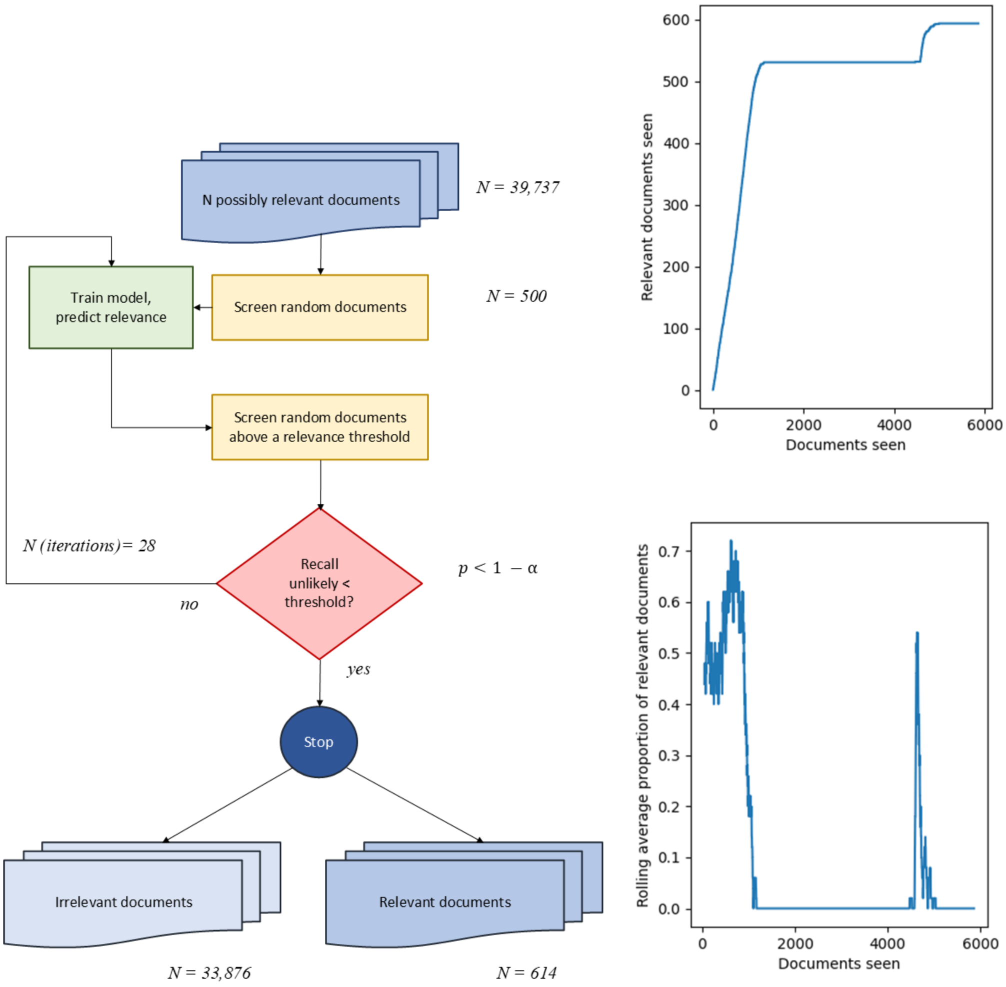

Supplementary Figure 3: Forest plot showing average effect size with 95% confidence intervals across primary studies. A forest plot is a graphical summary used in systematic reviews to display individual study estimates and the pooled effect. Study effects were harmonized by converting regression coefficients to correlation coefficients (r) based on sample size, then transformed to Fisher's Z (see Methods). Values of Z>0 indicate an increase in sales or market share of the more efficient appliance relative to less efficient alternatives.

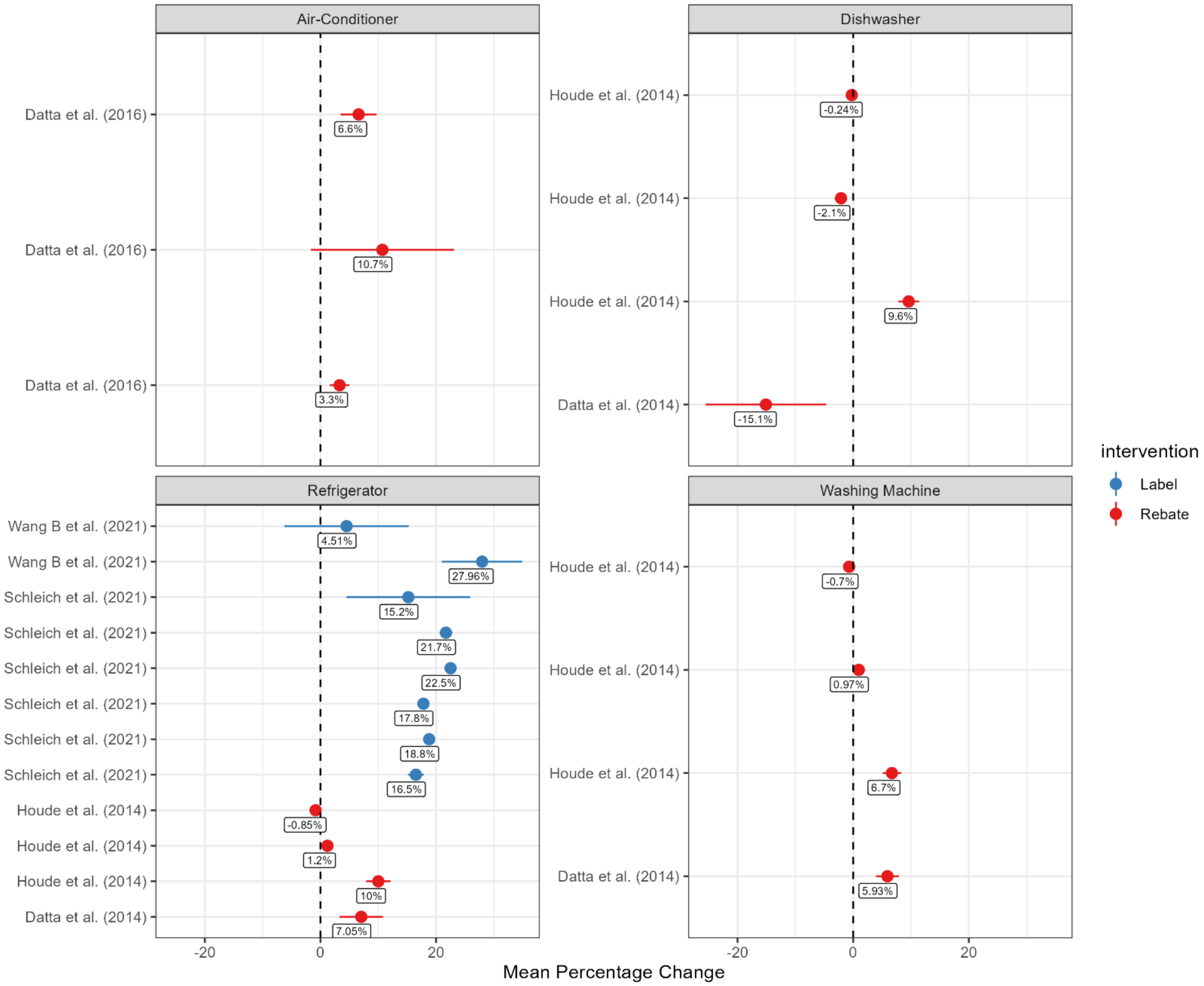

Supplementary Table 1: Search string applied to databases. Syntax is given for Web of Science Core Collection.

	Associated Keywords
**Population**	((household* OR home* OR housing OR dwelling* OR domestic OR domicil* OR residential OR residence) AND (applianc* OR "air-conditioning" OR "air conditioning" OR "air-condition*" OR "air condition*" OR HVAC OR heating OR "heat pump*" OR "heating ventilation" OR refrigerator OR fridge OR freezer OR "washing machine" OR "clothes washer" OR dishwasher* OR lighting OR "light bulb*" OR lightbulb* OR "tumble dryer*" OR "fridge freezer*" OR "heating system" OR "smart appliance*"))
… AND …	
**Intervention**	(purchase OR "purchase decision" OR adoption OR ((nudge OR nudging OR nudg*) NEAR/15 ("behavioral change" OR "behavioural change")) OR subsidy OR subsid* OR tax OR "tax credits" OR "utility rebate" OR rebate OR reward* OR credit OR "energy efficiency label" OR "eco-label*" OR label OR "eco label*"OR information OR "environmental certification" OR certificate OR "eco-certificat*" OR "eco certificat*" OR "cost disclosure" OR "information provision" OR "information provid*" OR feedback OR "real-time feedback" OR "social norm*" OR norm* OR "social influence" OR "social comparison" OR "social learning" OR "smart grid*" OR "demand side management" OR dsm OR "economic* intervention*" OR purchase OR installation OR cost* OR "window replacement" OR renovation* OR "heating system replacement" OR "energy-efficiency incentiv*" OR "stated preferences" OR "monetary incentive*" OR "non-monetary incentiv*" OR "non monetary incentiv*" OR "energy star" OR sale* OR "sales staff" OR "takeup" OR bonus OR "technology adoption" OR "behavioral change" OR "behavioural change")
**Comparator**	-
… AND …	
**Outcome**	((energy OR electric* OR gas) NEAR/15 (consumption OR conservation OR efficiency OR use OR demand OR usage))
**Study type**	-
**Note:** Since Publish or Perish does not allow for long search strings, a simplified query was run on this database: *(household OR home OR housing) AND (appliance OR appliances OR "air-conditioning" OR heating OR "heat pump" OR "heating ventilation" OR refrigerator OR fridge OR freezer OR "washing machine" OR "clothes washer" OR dishwasher OR lighting OR "tumble dryer" OR "fridge freezer" OR "heating system" OR "light bulb") AND (purchase OR "purchase decision" OR usage OR "usage decision") AND (incentive OR programme OR program OR label OR rebate OR feedback OR "tax credit" OR information)*.	

Supplementary Table 2: Inclusion/Exclusion criteria used for classifying studies

	Population	Intervention	Outcome	Study Type
**Inclusion**	Households; Individuals	Clear appliance specific intervention (i.e. labelling, feedback, information, monetary incentive); Command and control measures for appliances	Appliance purchase; WTP for energy efficiency; Energy consumption; Demand shifting	Empirical quantitative studies; Quasi-experimental studies; Choice experiments with real participants; Online experiments with real decisions (revealed preferences or self-reported behaviours); Market statistics studies; Stated preferences
**Exclusion**	Commercial /Industrial buildings; Grid-level agents; Automobiles	Dynamic pricing; Automatic demand control	Conveyance Awareness	Simulations; PoC; Qualitative studies; Case studies

We only tagged as relevant those studies that dealt with appliance purchases by households or individuals and/or contained information about specific appliance energy consumption in a household or dormitory. Further, studies tagged as relevant contained a quantitative estimate for appliance sales or energy saved through the effect of the relevant intervention. Studies that only provided an effect size but not the associated variance were not included in the final synthesis. In addition, studies where no obvious comparator group was available (untreated control group or pre-intervention data) or where the sample size was too small to extract meaningful estimates were excluded from the analysis. In particular, studies evaluating interventions such as Home Energy Reports or time-of-use pricing that directly target energy consumption of households were excluded because these studies do not isolate the contribution of energy-efficient appliances to changes in household energy use and have been synthesized extensively in prior reviews. Accordingly, studies focused on changes in energy consumption were included only when energy use was explicitly linked to appliance upgrades or replacement decisions (e.g., rebound effect analyses) following the application of behavioral, information or monetary interventions.

Supplementary Table 3: Overview of previously published related literature

Authors	Year	Methodology	Scope - Outcome	Scope - Intervention	Key Findings	# Included Studies (Effect Sizes)
Khanna et al.	2021	Systematic quantitative review	Energy usage	Information strategies, Economic incentives	Monetary incentives had a higher positive effect than information strategies. Some combinations of incentives provided significantly higher results than individual interventions, while others had about the same effect size.	122 (360)
Buckley, P.	2020	Systematic quantitative literature review	Energy usage	Informational and financial feedback	Interventions cause a 1.9–3.9% reduction in consumption, where individual and real-time feedback as well as personalized advice on how to save electricity are more effective than feedback on electricity costs and general electricity savings tips which lead to relative increases in consumption.	52 (128)
Labandeira, X., Labeaga, J. M., Linares, P., López-Otero, X.	2020	Systematic quantitative literature review	Energy usage and price of goods	standards, economic incentives, Information instruments	It is possible to achieve an 8–10% reduction in energy demand and a 7–9% increase in the price of durable goods through energy efficiency policies.	366 (1483)
Nemati, M. & Penn, J.	2020	Systematic quantitative literature review	Energy and water usage	Information-based interventions	Information-based interventions reduce consumption in the electricity sector by 6.48%.	116 (728)
Nisa, C. F., Bélanger, J.J., Schumpe, B. M., Faller, D. G.	2019	Systematic quantitative literature review	Energy (usage and purchase), transportation, water, and other mitigation-related behaviors	Behavioral interventions (appeals and nudges)	The impact on appliance adoption is estimated as only marginally significant, with the effect on energy usage only slightly higher.	83 (144)<br><br>Note: The Nisa et al. 2019 review includes 4 studies on appliance purchase, which are also included in this review.
Andor, M. A. & Fels, K. M.	2018	Systematic qualitative literature review	Energy usage	Non-price interventions: goal setting, labelling, social	While generally resulting in reduced energy consumption, the effects	44 (105)

Authors	Year	Methodology	Scope - Outcome	Scope - Intervention	Key Findings	# Included Studies (Effect Sizes)
				comparison, and commitment devices	significantly varied in size within an intervention.	
Laes, E., Mayeres, I., Renders, N., Valkering, P., Verbeke, S.	2018	Systematic qualitative literature review	Energy efficiency improvements (e.g. water heater, insulation, etc.)	Economic incentives, regulatory instruments, institutional frameworks, information measures.	A positive impact of financial and fiscal measures. The evidence on the relative impact of regulation vs. financial incentives is inconclusive. For information policies the quantitative evidence is still limited.	14
Wiese, C., Larsen, A. & Pade, L.-L.	2018	Unstructured qualitative literature review	Energy usage	Structural interventions, economic incentives, regulatory instruments, information and feedback, and commitment strategies	Some instrument interactions had a mitigating and others a reinforcing effect	Not provided
Šćepanović, S., Warnierb, M., Nurminena, J. K.	2016	Systematic qualitative literature review	Energy usage and purchase of efficient appliances	Information-based, gamification and monetary rewards, structural interventions	Efficacy of interventions varies greatly based on physical (environmental), socio-economic, cultural, and political contexts	189
Tsuda, K., Uwasu, M., Hara, K., Fuchigami, Y.	2016	Systematic qualitative literature review	Energy usage and peak shifting	Feedback	Demand control approaches such as dynamic pricing and information feedback either shift the peak load or reduce electricity consumption. In addition, the effectiveness of an instrument depends on the characteristics of the location, the household, the industry, and the climate.	110
Delmas, M. A., Fischlein, M., Asensio, O. I.	2013	Systematic quantitative literature review	Energy usage	Information strategies	On average, individuals in the experiments reduced their electricity consumption by 7.4%. Our results also show that strategies providing individualized audits and consulting are comparatively more	59 (156)

Authors	Year	Methodology	Scope - Outcome	Scope - Intervention	Key Findings	# Included Studies (Effect Sizes)
					effective for conservation behavior than strategies that provide historical, peer comparison energy feedback.	

Supplementary Table 4: Detailed descriptive statistics for all coded effect sizes

No	Variable	Stats / Values	Freqs (% of Valid)
1	Document Publication Year	Mean (sd) : 2016.6 (7)<br>min < med < max: 1982 < 2019 < 2025 IQR (CV) : 7 (0)	21 distinct values
2	Z_value	Mean (sd) : 0.1 (0.4)<br>min < med < max: -0.4 < 0 < 4 IQR (CV) : 0.1 (3.7)	292 distinct values
3	Total Sample Size	Mean (sd) : 19502.5 (107382.9)<br>min < med < max: 6 < 827 < 957080 IQR (CV) : 1363 (5.5)	113 distinct values
4	Appliance	• Air-Conditioner<br>• Clothes Dryer<br>• Dishwasher<br>• Heat Pump<br>• Lighting<br>• Refrigerator<br>• TV<br>• Washing Machine<br>• Water Heater	63 (17.9%)<br>19 (5.4%)<br>10 (2.8%)<br>29 (8.3%)<br>43 (12.3%)<br>99 (28.2%)<br>15 (4.3%)<br>27 (7.7%)<br>46 (13.1%)
5	Intervention	• Regulatory standards/Defaults<br>• Information<br>• Label<br>• Loan<br>• Rebate<br>• Subsidy	9 (2.6%)<br>55 (15.7%)<br>162 (46.2%)<br>12 (3.4%)<br>72 (20.5%)<br>41 (11.7%)
6	Continent	• Africa<br>• Asia<br>• Cross-country study<br>• Europe<br>• North and Central America<br>• Oceania	10 (2.8%)<br>111 (31.6%)<br>42 (12.0%)<br>92 (26.2%)<br>86 (24.5%)<br>10 (2.8%)
7	Study Design	• Control-treatment/Experimental<br>• DID<br>• PrePost<br>• Quasi-experimental<br>• Simulated counterfactual	105 (29.9%)<br>46 (13.1%)<br>30 (8.5%)<br>159 (45.3%)<br>11 (3.1 %)
8	Randomization	• No<br>• Yes	236 (67.2%)<br>115 (32.8%)
9	Stats Method	• OLS<br>• Means Differences<br>• Logit / Probit<br>• Other	124 (35.3%)<br>31 (8.8%)<br>180 (51.3%)<br>16 (4.6%)
10	Participant Type	• Homeowners<br>• Residents<br>• Unspecified	66 (18.8%)<br>133 (37.9%)<br>152 (43.3%)
11	Experimental Setting	• Household<br>• Online<br>• Retailer<br>• Lab	133 (37.9%)<br>92 (26.2%)<br>121 34.5%<br>5 ((1.4%))
12	Incentive Follow-through	• Revealed preference<br>• Self-reported behavior<br>• Stated preference	155 (44.7%)<br>65 (18.7%)<br>127 (36.6%)

No	Variable	Stats / Values	Freqs (% of Valid)
13	Controls Energy Prices		0 : 256 (72.9%)<br>1 : 95 (27.1%)
14	Controls Environmental Attitudes		0 : 255 (72.6%)<br>1 : 96 (27.4%)
15	Controls Demographics		0 : 133 (37.9%)<br>1 : 218 (62.1%)
16	Opted In	• No<br>• Yes	241 (68.7%)<br>110 (31.3%)
17	Aware of Study	• No<br>• Yes	160 (45.6%)<br>191 (54.4%)
18	Out of Sample Bias	• Probably no<br>• Probably yes	266 (75.8%)<br>85 (24.2%)
19	Reporting Biases	• Probably no<br>• Probably yes	314 (89.5%)<br>37 (10.5%)

Supplementary Table 5: Detailed descriptive statistics for effect sizes on purchase probability (included in the meta-analysis)

No	Variable	Stats / Values	Freqs (% of Valid)
1	Document Publication Year	Mean (sd) : 2015.8 (7.5)<br>min < med < max: 1982 < 2018 < 2024 IQR (CV) : 8 (0)	20 distinct values
2	Z_value	Mean (sd) : 0.1 (0.2)<br>min < med < max: -0.4 < 0 < 1.3 IQR (CV) : 0.1 (2)	207 distinct values
3	Total Sample Size	Mean (sd) : 2985.9 (8705.4)<br>min < med < max: 6 < 600 < 81095 IQR (CV) : 1020 (2.9)	80 distinct values
4	Appliance	• Air-Conditioner<br>• Clothes Dryer<br>• Dishwasher<br>• Heat Pump<br>• Lighting<br>• Refrigerator<br>• TV<br>• Washing Machine<br>• Water Heater	37 (14%)<br>19 (7.2%)<br>4 (1.5%)<br>21 (8%)<br>34 (12.9%)<br>76 (28.8%)<br>12 (4.5%)<br>18 (6.8%)<br>43 (16.3%)
5	Intervention	• Defaults<br>• Information<br>• Label<br>• Loan<br>• Rebate<br>• Subsidy	2 (0.8%)<br>53 (20.1%)<br>133 (50.4%)<br>12 (4.5%)<br>34 (12.9%)<br>30 (11.4%)
6	Continent	• Africa<br>• Asia<br>• Cross-country study<br>• Europe<br>• North and Central America<br>• Oceania	10 (3.8%)<br>81 (30.7%)<br>29 (11%)<br>84 (31.8%)<br>54 (20.5%)<br>6 (2.3%)
7	Study Design	• Control-treatment/Experimental<br>• DID<br>• PrePost<br>• Quasi-experimental<br>• Simulated counterfactual	101 (38.3%)<br>15 (5.7%)<br>27 (10.2%)<br>110 (41.7%)<br>11 (4.2%)
8	Randomization	• No<br>• Yes	152 (57.6%)<br>112 (42.4%)
9	Stats Method	• OLS<br>• Means Differences<br>• Logit / Probit<br>• Other	40 (15.2%)<br>31 (11.7%)<br>180 (68.2%)<br>13 (4.9%)
10	Participant Type	• Homeowners<br>• Residents<br>• Unspecified	41 (15.5%)<br>103 (39%)<br>120 (45.5%)
11	Experimental Setting	• Household<br>• Online<br>• Retailer<br>• Lab	106 (40.2%)<br>89 (33.7%)<br>64 (24.2%)<br>5 (1.9%)
12	Incentive Follow-through	• Revealed preference<br>• Self-reported behavior<br>• Stated preference	91 (34.5%)<br>46 (17.4%)<br>127 (48.1%)
13	Controls Energy Prices		0 : 207 (78.4%)

No	Variable	Stats / Values	Freqs (% of Valid)
			1 : 57 (21.6%)
14	Controls Environmental Attitudes		0 : 192 (72.7%)<br>1 : 72 (27.3%)
15	Controls Demographics		0 : 96 (36.4%)<br>1 : 168 (63.6%)
16	Opted In	• No<br>• Yes	176 (66.7%)<br>88 (33.3%)
17	Aware of Study	• No<br>• Yes	87 (33.0%)<br>177 (67.0%)
18	Out of Sample Bias	• Probably no<br>• Probably yes	188 (71.2%)<br>76 (28.8%)
19	Reporting Biases	• Probably no<br>• Probably yes	233 (88.3%)<br>31 (11.7%)

Supplementary Table 6: Results from meta-regression on purchase probability

Variable	REML-Multilevel		Corrected for publication bias		Excl. outliers based on Cook's d and corrected for publication bias	
	Estimated beta coefficient	p-value	Estimated beta coefficient	p-value	Estimated beta coefficient	p-value
**Intervention type**						
Information	0.07**	0.01	0.06*	0.03	0.06***	0.00
Label	0.12***	0.00	0.10***	0.00	0.09***	0.00
Loan	- 0.06	0.33	- 0.06	0.30	- 0.03	0.47
Rebate	0.12***	0.00	0.11**	0.00	0.08**	0.00
Subsidy	0.11**	0.02	0.10*	0.02	0.10***	0.00
z-variance			2.95**	0.02	4.44***	0.00
**Appliance**						
Air-Conditioner	0.02	0.68	0.01	0.81	0.07**	0.02
Clothes Dryer	0.11**	0.03	0.08	0.11	0.06*	0.09
Dishwasher	0.09	0.30	0.07	0.41	0.04	0.51
Heat Pump	0.04	0.55	0.04	0.55	0.03	0.44
Lighting	0.11***	0.01	0.09**	0.03	0.09***	0.00
Refrigerator	0.18***	0.00	0.16***	0.00	0.10***	0.00
TV	0.09	0.17	0.07	0.31	0.06	0.21
Washing Machine	0.12***	0.00	0.10***	0.00	0.07**	0.02
Water Heater	0.06	0.18	0.05	0.21	0.06*	0.08
z-variance			3.27**	0.01	4.79	0.00

*Note: The dependent variable is the standardized Fisher's Z calculated for each effect size reported by studies. Z > 0 indicates a higher willingness to pay for energy efficient appliances. ***p <= 0.01; **p <= 0.05; *p <= 0.10.*

Supplementary Table 7: Results from the heterogeneity analysis using Bayesian model averaging

	PIP	Post Mean	Post SD	Cond.Pos.Sign	Idx
z_variance	1.00	2.65	0.43	1.00	1.00
incentive_follow_through_stated_preference	1.00	0.16	0.03	1.00	23.00
participant_type_residents	1.00	- 0.12	0.03	-	28.00
rebate_0	1.00	- 0.15	0.04	-	4.00
appliance_Refrigerator	0.98	0.13	0.03	1.00	9.00
appliance_Lighting	0.98	0.14	0.05	1.00	8.00
reportingBiases_probably_yes	0.82	0.09	0.06	1.00	33.00
randomisation_dummy_0	0.50	- 0.03	0.04	0.00	31.00
subsidy_1	0.50	0.06	0.06	1.00	5.00
appliance_Washing_Machine	0.48	0.05	0.06	1.00	11.00
incentive_follow_through_self-reported_behavior	0.46	0.05	0.06	1.00	22.00
study_design_quasi-experimental	0.33	0.02	0.03	1.00	18.00
appliance_Clothes_Dryer	0.24	0.02	0.05	1.00	14.00
appliance_Heat_Pump	0.19	- 0.02	0.04	0.00	10.00
controls_demographics_0	0.13	0.01	0.02	0.99	36.00
appliance_TV	0.13	- 0.01	0.04	0.01	13.00
statistical_technique_OLS	0.13	- 0.01	0.02	0.00	15.00
participant_type_homeowners	0.08	- 0.01	0.02	0.02	27.00
experimental_setting_household	0.08	0.00	0.01	0.95	24.00
statistical_technique_diff_of_means	0.08	- 0.00	0.02	0.03	16.00
loan_1	0.07	- 0.00	0.02	0.00	6.00
controls_environmental_attitudes_0	0.06	0.00	0.01	0.98	35.00
experimental_setting_lab	0.06	- 0.01	0.03	0.00	26.00
aware_of_study_1	0.06	0.00	0.01	0.99	29.00
experimental_setting_online	0.05	- 0.00	0.01	0.06	25.00
study_design_simulated_counterfactual	0.05	- 0.00	0.02	0.07	21.00
controls_energy_prices_1	0.05	0.00	0.01	0.96	34.00
outOfSampleBias_probably_yes	0.05	0.00	0.01	0.86	32.00
appliance_Air-Conditioner	0.05	- 0.00	0.01	0.15	7.00
appliance_Dishwasher	0.04	0.00	0.02	0.99	12.00
opt_in_1	0.04	0.00	0.01	0.84	30.00
label_1	0.04	0.00	0.01	0.92	3.00
study_design_DID	0.04	0.00	0.01	0.96	19.00
information_1	0.03	0.00	0.01	0.94	2.00
study_design_pre_and_post	0.03	- 0.00	0.01	0.39	20.00
statistical_technique_Other	0.03	0.00	0.01	0.90	17.00
(Intercept)	1.00	0.11	NA	NA	-

Note: The dependent variable is the standardized Fisher's Z calculated for each effect size reported by studies. Z > 0 indicates a higher willingness to pay for energy efficient appliances. The posterior inclusion probability (PIP) indicates the relevance of each variable. Commonly, variables with a PIP above 0.5 are interpreted to be relevant explanatory factors, while variables with lower PIPs are unable to capture the observed heterogeneity. The table furthermore provides the posterior mean and standard deviation of the estimated effect averaged across all meta-regressions that include the respective variable.

Supplementary Table 8: Studies that measure energy consumption post treatment

	Study	Appliance	Increase/Decrease in energy consumption	Study design	Energy use measurement	Year data	Location	Measurement level	Intervention
1	Alberini and Towe (2015)	1. General<br>2. Heat pumps	Energy consumption reduces by ~5% for both energy audits and heat pump rebates, inclusive of any potential rebound effects.	Quasi-experiment (matching), triple differences	Actual electricity consumption measured by utility	2008-2012, one year post intervention	Maryland, US	Total	1. Information (Home audits)<br>2. Rebates for heat pumps<br>No general information or feedback on electricity consumption
2	Alberini, Gans & Towe (2016)	Heat Pumps	8% reduction in electricity consumption on average on adoption of a more efficient heat pump. 16% reduction without incentives, no reduction with incentives	Quasi-experiment, difference-in-difference estimation	Actual electricity consumption measured by utility	2011	Maryland, US	Total	Mandatory technology standards, Monetary interventions
3	Cheng, Zhang & Cai (2025)	Air conditioners, Refrigerators, dishwashers and TVs	Efficient labeled appliances (except refrigerators) are correlated with higher use and energy consumption as compared to unlabeled appliances	Surveys, ordinary least squares with dummy variables	Unclear	2015	China	Total	Energy efficiency labeling
4	Chuang, Delmas, & Pincetl (2022)	Pool pumps, refrigerators, air conditioners, dishwashers and clothes washers	• 4% reduction in total electricity use<br>• 12%–13% reduction with pool pumps and 6% reduction refrigeration<br>• 1% reduction in air conditioner upgrades<br>• 0.3% to 0.7% with lighting<br>• Zero savings with dishwashers and clothes washers	Quasi-experiment, difference-in-difference estimation with matching	Actual electricity consumption measured by utility	2010-2015	Southern California, USA	Total	Subsidies for purchase more efficient appliances
5	Chun and Jiang (2013)	Lighting	10% increase in lighting efficiency results in about 6.5–7.7% in energy savings inclusive of rebound effect. The rebound effect led to erosion in savings by 23-35%.	Survey, in home inspection, OLS/Instrument variable	Self-reported electricity consumption	2009	Pakistan	Lightning	Information and subsidies for adoption of compact fluorescent lamps (CFL)

	Study	Appliance	Increase/Decrease in energy consumption	Study design	Energy use measurement	Year data	Location	Measurement level	Intervention
6	Davis, Fuchs & Gertler (2014)	1. Refrigerators<br>2. Air conditioners	1. 8% reduction in electricity consumption of refrigerators<br>2. Increase or no change in electricity consumption of air conditioners	Quasi-experiment (matched samples), difference-in-difference estimation	Actual electricity consumption measured by utility	2009-2012	Mexico	Total	Monetary interventions for replacement of inefficient appliances, along with information awareness campaigns
7	Hammerle and Burke (2022)	Hot water heaters	Net reduction in energy consumption of households with more efficient heating. Rebound effect not quantified.	Observational, panel fixed effects model	Actual electricity consumption measured by utility	2015-2020	Australian Capital territory	Total	Rebates for replacing natural gas heaters with more efficient electric ones
8	Houde and Aldy (2017)	Refrigerators, dishwashers and clothes washers	Short term reduction of 0.35% for refrigerators, 1.7% for clothes washers and null for dishwashers. Over the long term, the effect converges to zero for all three appliances	Quasi-experiment, difference-in-difference estimation	Actual electricity consumption measured by utility	2008-2012	US, nationwide	Total	Rebates for appliances labeled as energy efficient
9	Liddle, Loi, Owen, Tao (2020)	Air-conditioner	7.8% reduction in energy consumption after the purchase of a more efficient AC inclusive of rebound effect. Rebound effect led to erosion in savings by 38.2%.	Quasi-experiment, difference-in-difference estimation	Actual electricity consumption measured by utility	2014-2017	Singapore	Total	Labeling & Mandatory Energy Performance Standards
10	Mizobuchi, Takeuchi (2016)	Air conditioners	Energy consumption of households with efficient ACs was 8.9% lower than households with inefficient units	Quasi-experiment	Actual electricity consumption measured by utility	2011-2012	Japan	Total	Awareness campaign, incentives for replacement of inefficient appliances, mandatory technology standard
11	Naeher et al. (2024)	Lighting	Decrease in the amount paid for electricity (implying a reduction in energy consumption)	Quasi-experiment, difference-in-	Self-reported average cost of energy consumption	2009-2014	Mexico	Total	Free replacement of incandescent bulbs with efficient CFL, along with

	Study	Appliance	Increase/Decrease in energy consumption	Study design	Energy use measurement	Year data	Location	Measurement level	Intervention
				difference estimation					information awareness campaigns
12	Shojaeddini and Gilbert (2023)	Lighting	• Reduction in energy consumption across all households (no “backfire” effect)<br>• Rebound effects are larger for low-income households and those in smaller homes	Quasi-experiment, instrument variable and matching	Self reported hours of lighting use during a summer day and imputed electricity consumption	2009	USA	Appliance level	Subsidies for efficient lighting upgrades
13	Sun (2018)	1. Dishwasher, 2. Air-conditioner	1. Decrease in frequency of use, energy consumption associated with having an efficient dishwasher.<br><br>2. No change in frequency of use, reduction in energy consumption associated with an efficient air-conditioner.	Quasi-experiment, instrument variable estimation	Self-reported frequency of use via survey, annual electricity consumption	2009	US, nationwide	Appliance /space cooling	Labeling (Energy Star program in the US)
14	Yao, Liu & Yan (2014)	Not specified	Significant rebound effects that increase overall household electricity consumption	Surveys, quantile regression	Unclear	2013	China	Total	Subsidy for energy efficient appliances

Supplementary Table 9: Complete list of studies included in the analysis[1]

Note: Complete reference to papers included in the document below can be found here.

	Author	Year	Title
Usage			
1	Alberini, Gans, Towe	2016	Free Riding, Upsizing, and Energy Efficiency Incentives in Maryland Homes
2	Alberini, Towe	2015	Information v. energy efficiency incentives: Evidence from residential electricity consumption in Maryland
3	Cheng, Zhang, Cai	2025	Appliance energy efficiency policies and electricity consumption: Evidence from China
4	Chuang, Delmas, Pincetl	2022	Are Residential Energy Efficiency Upgrades Effective? An Empirical Analysis in Southern California
5	Chun, Jiang	2013	How households in Pakistan take on energy efficient lighting technology
6	Davis, Fuchs, Gertler	2014	Cash for Coolers: Evaluating a Large-Scale Appliance Replacement Program in Mexico
7	Hammerle, Burke	2022	From natural gas to electric appliances: Energy use and emissions implications in Australian homes
8	Houde, Aldy	2017	Consumers' Response to State Energy Efficient Appliance Rebate Programs
9	Liddle, Loi, Owen, Tao	2020	Evaluating consumption and cost savings from new air-conditioner purchases: The case of Singapore
10	Mizobuchi, Takeuchi	2016	Replacement or additional purchase: The impact of energy-efficient appliances on household electricity saving under public pressures
11	Naeher, Narayanan, Ziulu	2024	Cash for Coolers or Sustainable Lighting? Assessing Different Components of a Large-Scale Energy Efficiency Program in Mexico
12	Shojaeddini, Gilbert	2023	Heterogeneity in the Rebound Effect: Evidence from Efficient Lighting Subsidies
13	Sun	2018	Heterogeneous direct rebound effect: Theory and evidence from the Energy Star program
14	Yao, Liu, Yan	2014	A quantile approach to assess the effectiveness of the subsidy policy for energy-efficient home appliances: Evidence from Rizhao, China
Market share			
15	Buettner, Madzharova	2024	Promoting Sales of Energy Efficient Household Appliances: Outcomes and Cost-Effectiveness of Rebate Programs
16	Datta, Filippini	2016	Analysing the impact of ENERGY STAR rebate policies in the US
17	Datta, Gulati	2014	Utility rebates for ENERGY STAR appliances: Are they effective?
18	Houde, Aldy	2017	Consumers' Response to State Energy Efficient Appliance Rebate Programs
19	Schleich, Durand, Brugger	2021	How effective are EU minimum energy performance standards and energy labels for cold appliances?

[1] Houde and Aldy (2017) is included in both market share and usage papers as it has information on both aspects

	Author	Year	Title
20	Wang B, Deng, Liu, Sun, Wang ZH	2021	Effect of energy efficiency labels on household appliance choice in China: Sustainable consumption or irrational intertemporal choice?
Purchase			
21	Alberini, Bigano, Boeri	2014	Looking for free riding: energy efficiency incentives and Italian homeowners
22	Allcott, Sweeney	2017	The Role of Sales Agents in Information Disclosure: Evidence from a Field Experiment
23	Allcott, Taubinsky	2015	Evaluating Behaviorally Motivated Policy: Experimental Evidence from the Lightbulb Market
24	Anderson, Claxton	1982	Barriers to Consumer Choice of Energy Efficient Products
25	Andor, Gerster, Goette	2019	How effective is the European Union energy label? Evidence from a real-stakes experiment
26	Asinyaka	2019	Willingness to Pay for Energy Efficient Refrigerating Appliances in Accra, Ghana: A Choice Experiment Approach
27	Caldwell, Vaughn, Harrod E, Harrod J	2019	Social Marketing-Enhanced Home Energy Education Encourages Adoption of Energy-Saving Practices
28	Ceolotto, Denny	2024	Putting a New 'Spin' on Energy Information: Measuring the Impact of Reframing Energy Efficiency Information on Tumble Dryer Choices in a Multi-country Experiment
29	Davis, Metcalf	2016	Does Better Information Lead to Better Choices? Evidence from Energy-Efficiency Labels
30	DECC	2014	Evaluation of the DECC/John Lewis energy labelling trial
31	del Mar Sola, de Ayala, Galarraga	2021	The Effect of Providing Monetary Information on Energy Savings for Household Appliances: A Field Trial in Spain
32	del Mar Sola, Escapa Galarraga	2023	Effectiveness of monetary information in promoting the purchase of energy-efficient appliances: Evidence from a field experiment in Spain
33	Denny	2022	Long-term Energy Cost Labelling for Appliances: Evidence from a Randomised Controlled Trial in Ireland
34	Deutsch	2010	The effect of life-cycle cost disclosure on consumer behavior: evidence from a field experiment with cooling appliances
35	Dieu-Hang, Grafton, Martinez-Espineira, Garcia-Valinas	2017	Household adoption of energy and water-efficient appliances: An analysis of attitudes, labelling and complementary green behaviours in selected OECD countries
36	Faure, Guetlein, Schleich	2021	Effects of rescaling the EU energy label on household preferences for top-rated appliances
37	Figueroa, de Moliere, Pegels, Never, Kutzner	2019	Show me (more than) the money! Assessing the social and psychological dimensions to energy efficient lighting in Kenya
38	Galarraga, Heres, Gonzalez, Eguino	2011	Price premium for high-efficiency refrigerators and calculation of price-elasticities for close-substitutes: a methodology using hedonic pricing and demand systems
39	Galarraga, Gonzalez-Eguino, Markandya	2011	Willingness to pay and price elasticities of demand for energy-efficient appliances: Combining the hedonic approach and demand systems
40	Gao, Tavoni	2024	Forget-Me-Not: The Persistent Effect of Information Provision for Adopting Climate-Friendly Goods

	Author	Year	Title
41	Goto H, Goto M, Sueyoshi	2011	Consumer choice on ecologically efficient water heaters: Marketing strategy and policy implications in Japan
42	Hafner, Elmes, Read, White	2019	Exploring the role of normative, financial and environmental information in promoting uptake of energy efficient technologies
43	Harajli, Chalak	2019	Willingness to Pay for Energy Efficient Appliances: The Case of Lebanese Consumers
44	Heinzle, Wüstenhagen	2012	Dynamic Adjustment of Eco-labeling Schemes and Consumer Choice - the Revision of the EU Energy Label as a Missed Opportunity?
45	Jain, Rao, Patwardhan	2018	Consumer preference for labels in the purchase decisions of air conditioners in India
46	Jeong, Kim	2015	The effects of energy efficiency and environmental labels on appliance choice in South Korea
47	Kallbekken, Saelen, Hermansen	2013	Bridging the Energy Efficiency Gap: A Field Experiment on Lifetime Energy Costs and Household Appliances
48	Kuhn, Thøgersen, Kutzner	2023	No trust in the choice architect? No problem! On the minor role of trust for the effectiveness of default interventions promoting the choice of energy-efficient appliances
49	Li, Cao	2021	Effectiveness of China's Labeling and Incentive Programs for Household Energy Conservation and Policy Implications
50	Li, Clark, Jensen, Yen	2014	The Effect of Mail-in Utility Rebates on Willingness-to-Pay for ENERGY STAR® Certified Refrigerators
51	Liu, Jin	2019	Visualisation approach and economic incentives toward low carbon practices in households: A survey study in Hyogo, Japan
52	London Economics	2014	Study on the impact of the energy label – and potential changes to it – on consumer understanding and on purchase decisions
53	Ma, Yu, Urban	2018	Green transition of energy systems in rural China: National survey evidence of households' discrete choices on water heaters
54	Mekonnen, Hassen, Jaime, Toman, Zhang	2023	The effect of information and subsidy on adoption of solar lanterns: An application of the BDM bidding mechanism in rural Ethiopia
55	Nakano, Zusman, Nugroho, Kaswanto, Arifin N, Munandar, Arifin HS, Muchtar, Gomi, Fujita	2018	Determinants of energy savings in Indonesia: The case of LED lighting in Bogor
56	Neves, Oliveira	2021	Drivers of consumers' change to an energy-efficient heating appliance (EEHA) in households: Evidence from five European countries
57	Newell, Siikamäki	2014	Nudging Energy Efficiency Behavior: The Role of Information Labels
58	Nishijima, Kagawa, Nansai, Oguchi	2019	Effects of product replacement programs on climate change
59	Orset	2021	Is information a good policy instrument to influence the energy behaviour of households?

	Author	Year	Title
60	Razali, Kamaludin, Azlina	2022	Consumer Preference for Energy Label in the Purchase Decision of Refrigerator: A Discrete Choice Experiment Approach in the East Coast, Malaysia
61	Revelt, Train	1998	Mixed Logit with Repeated Choices: Households' Choices of Appliance Efficiency Level
62	Sammer, Wüstenhagen	2006	The influence of eco-labelling on consumer behaviour – results of a discrete choice analysis for washing machines
63	Schultz, Colehour, Vohr, Bonn, Bullock, Sadler	2015	Using Social Marketing to Spur Residential Adoption of ENERGY STAR®-Certified LED Lighting
64	Shen, Saijo	2009	"Does an energy efficiency label alter consumers' purchasing decisions? A latent class approach based on a stated choice experiment in Shanghai"
65	Shen, Liu,Patwardhan	2022	The Effect of Rebate and Loan Incentives on Residential Heat Pump Adoption: Evidence from North Carolina
66	Skourtos, Damigos, Tourkolias, Kontogianni	2021	Efficient energy labelling: the impact of information content and style on product choice
67	Stadelmann, Schubert	2018	How Do Different Designs of Energy Labels Influence Purchases of Household Appliances? A Field Study in Switzerland
68	Train, Atherton	1995	REBATES, LOANS, AND CUSTOMERS CHOICE OF APPLIANCE EFFICIENCY LEVEL - COMBINING STATED AND REVEALED-PREFERENCE DATA
69	Wang	2023	Impact of incentives to purchase energy-efficient products: evidence from Chinese households based on a mixed logit model
70	Ward, Clark, Jensen, Yen, Russell	2011	Factors influencing willingness-to-pay for the ENERGY STAR (R) label
71	Wasi, Carson	2013	The influence of rebate programs on the demand for water heaters: The case of New South Wales
72	Zha, Yang, Wang, Zhou	2020	Appliance energy labels and consumer heterogeneity: A latent class approach based on a discrete choice experiment in China
73	Zhou, Bukenya	2016	Information inefficiency and willingness-to-pay for energy-efficient technology: A stated preference approach for China Energy Label

Supplementary Table 10: Average effect size by each study design employed

We have calculated average effect size by each study design employed and have reported the results in the Supplementary Table 10. The results are also reproduced below. Note that while there is difference in the effect size, the confidence intervals overlap and adding the variable Study_design as a moderator variable in the meta-regression shows that there is no statistical difference between the various study designs.

study_design	k	estimate	se	zval	pval	ci.lb	ci.ub
control_&_treatment	101	0.10	0.03	3.28	0.00	0.04	0.16
DID	15	0.11	0.01	18.36	0.00	0.10	0.12
pre_and_post	27	0.10	0.05	1.94	0.05	-	0.19
quasi-experimental	100	0.10	0.02	4.87	0.00	0.06	0.15
simulated_counterfactual	11	0.01	0.01	0.99	0.32	- 0.01	0.04

Supplementary Table 11: Codebook used to record necessary information from the included studies

	Field Name	Explanation	Choices or Examples
1	coder	Initials of the coder	SR – Sangeeth Raja
2	sr_no	Serial number, to identify the number of effect sizes	1,2,3,4
3	study_ID	To identify unique studies, the code starts with 1000, so if a single study has multiple effect sizes the study ID should be the same for all the rows.	100<br>101<br>102
4	authors	Authors of the paper	Houde & Aldy
5	year_publication	The year of publication of the study	2014
6	doc_title	Title of the Paper	…
7	abstract	Text of the Abstract	…
8	study_type	Whether Study looked at usage of appliance or purchase/replacement of appliance	usage – 1<br>purchase - 2
9	year_data_<br>study	Fill in the years the data from the study was collected	1990-2009<br>2009<br>NA
10	exclusion_<br>reason	Explain if you decided to exclude the study<br>If the study is included - fill in NA	- simulation<br>- PoC<br>- dep_var<br>- quality<br>- case study<br>- intervention<br>- no appliance breakdown<br>- other<br>- NA
11	page	Page of the study where results were found, if multiple pages - mention multiple pages.<br>If no page number is given, then give the PDF page number.	13
12	statistical_technique	Studies employ different techniques to estimate effects. We are interested in the type of model employed to estimate the delta in the original study.<br><br>A non-exhaustive list is mentioned in the examples. If other models or techniques used not mentioned in the list add it to the code sheet drop down options and discuss in the group<br><br>Note: Don’t code Difference in Difference as effect statistical technique. Here we just capture the type of regression and let Difference in Difference be the study design	- linear probability model<br>- logit/probit<br>- RDD<br>- OLS<br>- propensity score matching<br>- IV<br>- diff of means<br>- ANOVA<br>- logit<br>- 2SLS<br>- negative binomial count mode<br>- ITT
13	study_design	The study design captures the setting in which the experiment was conducted. An exhaustive list is provided in the examples.	- control & treatment<br>- pre and post<br>- diff-in-diff<br>- quasi-experimental<br>- correlation

14	country	The country where the experiment was conducted, or data was collected.	US, Germany, UK
	Risk of Bias Assessment		
15	incentive_follow_through	Whether participants must commit to their decision.	- revealed preference<br>- stated preference<br>- self-reported behaviour<br>- NA
16	experimental_setting	Was the experiment carried out in a retail store, directly in households, online, in labs, through survey or other unique experimental settings.	- retailer<br>- online<br>- lab<br>- sales agent<br>- household
17	participant_type	What can we describe the participants as.	- students<br>- store visitors<br>- homeowners<br>- tenants<br>- callers<br>- residents<br>- individuals<br>- unclear<br>- other
18	aware_of_study	Whether participants are aware they are part of a study or not (at the moment of making the purchasing decision).	1 – yes<br>0 – no
19	opt_in	Were households first selected and then allowed to opt out of the intervention or were the households required to opt-in to the intervention or neither<br><br>Note: Write **opt_in** when households are given the option to opt-into the experiment explicitly.	- opt_in<br>- opt_out<br>- no such option
20	randomisation_dummy	Were participants randomly assigned to control and treatment group and/or between different treatment groups?	1 – yes<br>0 – no
21	outOfSampleBias	Were the control and treatment group not representative of the average population of the corresponding area?<br><br>Choose probably yes if the sample is not representative and there probably is an out of sample bias. If participants can self-select into treatment choose probably yes.<br>**Choose probably no if the authors convincingly argue and show that their sample is representative.**	- probably yes<br>- probably no<br>- unclear
22	reportingBiases	Was there bias as to which effect sizes (outcome reporting bias) or which estimates (analysis reporting bias) are reported by the authors?<br><br>**If the paper manages to convince you that they have done a good job, choose probably no.**	- probably yes<br>- probably no<br>- unclear
23	notes_bias	If bias reported – elaborate with text in the notes column.	…
	**Intervention Type**	All dummy variables below: if this intervention is used in the study, then mark it as 1, if not - 0	

24	feedback	Code as 1 if the study estimates the impact of feedback as a policy instrument, whereby the household is getting updates on their electricity usage in relation to specific appliance(s) in or without comparison to peers.	1/0
25	information	Code as 1 if the study estimates the impact of information as a policy instrument, whereby the household is getting information on the environmental or other consequences of high energy intensity of appliances, or information on potential savings associated with appliance upgrade.	1/0
26	label	Code as 1 if the study estimates the impact of label as a policy instrument, whereby appliances get different energy efficiency labels in the retail stores or online.	1/0
27	rebate	Code as 1 if the study estimates the impact of rebate as a policy instrument, whereby the household pays the full amount to the retailer, and gets a specific proportion of it back either immediately or with a delay.	1/0
28	tax_credit	Code as 1 if the study estimates the impact of tax credit as a policy instrument, whereby a household gets a tax reduction equal to the amount of or a proportion of the amount of an efficient appliance.	1/0
29	subsidy	Code as 1 if the study estimates the impact of subsidy as a policy instrument, whereby the household has to pay only a reduced price to the retailer.	1/0
30	loan	Code as 1 if the study estimates the impact of loan as a policy instrument, whereby the household gets a loan on the amount of a more efficient appliance.	1/0
31	choice_architecture	Code as 1 if the study estimates the impact of choice architecture as a policy instrument, whereby the design of choice presentation to the decision-maker is altered.	1/0
32	command_control	Code as 1 if the study estimates the impact of command and control as a policy instrument, such as government-defined energy standards. Etc	
33	notes_intervention	For the intervention coding – describe here any specific circumstances (e.g. when multiple interventions are applied at the same time or one after another and/or we can't differentiate specific effect sizes for each).	…
	Appliance Type	All dummy variables below: if this appliance is in the study, then mark it as 1, if not - 0	
34	washing_ machine		1/0
35	clothes_dryer	Also tumble dryer, etc.	1/0
36	dishwasher		1/0
37	refrigerator	We will include freezers in this as well.	1/0
38	heat_pump		1/0
39	lighting		1/0
40	water_heater		1/0
41	ac		1/0
42	tv		1/0
43	notes_dep_var	Use these notes section to mention specifics of the dependent variables or any unique circumstances in the original study when it comes to the dependent variable	…

44	dep_var purchase_category	What is the dependent variable when the study is looking at purchase of appliances. The list in the examples is an exhaustive list of categories.	- market share of energy efficient appliance<br>- purchase probability of efficient appliance<br>- WTP for efficient appliance<br>- energy use after switching to a more efficient appliance
45	dep_var_original_study	Write out explicitly what the exact dependent variable is in the original study not just from the methods section of the study but also from the interpretation of results that you are reporting.	Record the original phrasing for the dependant variable from the study
46	dep_var_transformation	Has the dep variable been transformed to log scale or other transformations & describe the interpretation of the dep variable as given in original study	dummy (0/1)<br>log<br>other
47	coefficient	Value of the relevant regression coefficient in absolute value.<br>NA if not available	
48	effect_direction	Capture the direction of the effect of the intervention.<br><br>We are interested in promoting energy efficient appliances so the increase/decrease should be relative to that.<br><br>In terms of energy usage, intuitively, it's the other way around: increase means more energy was used, decrease means less energy was used.	- increase<br>- decrease<br>- NA
49	coefficient_se	The variance is captured by the standard error of the coefficient.<br><br>NA if not available	
	**Significance Variables**	If a study provides the coefficient and standard error, calculate the specific t-statistic (β/se) and use that to calculate a corresponding P-value.<br><br>If a study only provides the level of significance (no standard error, no t-statistic, no specific p-value) then capture the level of significance in the p-value field and calculate the t-statistic using the level of significance and mark it as a lower bound (if significant) or higher bound (if not significant)	
50	p_value		0.1
51	significance_bound		1 - lower bound<br>2 - upper bound<br>3 – actual
52	t_stat	Record t-statistic in absolute values.	…
53	percent_change	If the effect or delta is reported in percent change, then report the value here.<br>**We report the % change only when there is no coefficient reported.**	…
	**Control & Treatment (Difference of means)**	Capture both the control and treatment group means where possible. Otherwise capture the difference along with the value of the relevant test statistic (t, Chi or F).	

54	control_mean		
55	control_sd		
56	treated_mean		
57	treated_sd		
58	diff_mean		
59	pooled_sd		
	Sample Sizes (all statistical techniques)	Record the sample sizes for different groups here.	
60	control_sample_size	Record the size of the control group. There is possibly only one control group for multiple treatments.	
61	treatment_sample_size	Capture sample size for specific effect being captured (this should correspond to one treatment or combination of treatments)	
62	total_sample_size	Record the full sample site, including control and all relevant treatments.	
63	total_observations	Record the number of total observations.	
	Moderator Variables		
64	monetary_incentive_size	For all of the monetary incentives (rebate, subsidy, tax credit, loan): record here the size of the incentive (if reported).	$ (US dollar)<br><br>NA
65	monetary_incentive_relative	For all of the monetary incentives (rebate, subsidy, tax credit, loan): record here the relative size of the incentive (if reported) to the price of the appliance.	…%<br><br>NA
66	monetary_incentive_timeframe	For all of the monetary incentives (rebate, subsidy, tax credit, loan): record here whether the payment was upfront or claimed post purchase.	- current<br>- delayed<br>- NA
67	label_monetary	Does the label mention the money you're going to be saving if you buy this appliance	1 – yes<br>0 - no
68	label_monetary_size	If yes how much? (per annum, if specified otherwise – convert)	USD $
69	label_consumption	Does the label mention the savings in electricity in buying the appliance	1 – yes<br>0 - no
70	label_consumption_size	If yes how much with units? (kWh/annum)	KWh / annum
71	label_scale	Does the label have a scale showing the appliance relative to other appliances<br>For Eg: A scale of A+++, A++, B+, C or<br>A scale from 1 - 5 or<br>A scale A+,B+,C<br><br>If the labelling scheme uses any of scaling like the ones mentioned above record that as a yes	- numerical<br>- alphabetical<br>- alphabetical with +<br>- binary<br>- other
	Control Definition	List the controls which are being used to better isolate the effect of the intervention. Which elements that might affect energy consumption (besides the variable of interest (intervention)) are included in the model. These are all dummy variables (code 1/0 based on whether included or not).	

72	controls energy prices		0/1
73	controls environmental attitudes		0/1
74	controls demographics		0/1

Supplementary Notes

Supplementary Note 1: Ecosystem of Reviews for understanding reduction in energy consumption

The interventions used in the studies were classified into information strategies (information provision, label, audits); behavioral interventions (choice architecture, feedback); and monetary interventions (tax credits, rebates, grants) following previous studies in energy use among households [1–3]. This classification has been adopted across a series of reviews and meta-analyses that form an "ecosystem of reviews" that evaluates interventions in households' consumption choices in food, transport and buildings. This series of coordinated systematic reviews explores the effectiveness of demand-side interventions to induce behavioral change in key GHG emissions sectors, including food [4], transport [5], and buildings 1. All reviews are based on a set of harmonized inclusion criteria and screening methodology.

Supplementary Note 2: Publication Bias

Publication bias has been detected previously in the literature on experiments involving household energy consumption more generally[3]. Publication bias results in a correlation between standard error and effect size. The correlation can occur due to two reasons. First, researchers (or editors or referees) may prefer statistically significant results. Researchers working with small sample sizes and large standard errors may try, for instance, different combinations of control variables until they obtain an estimate large enough to offset the standard error. Second, researchers may prefer an intuitive sign of the estimates and discard those with the opposite sign. The association between standard error and effect size can therefore be used to detect and correct publication bias.

It is helpful to evaluate the relationship visually using the so-called funnel plot: a scatter plot with the effect sizes on the horizontal and their standard errors on the vertical axis. Based on the intuition described in the previous paragraph, an asymmetry of the funnel plot suggests publication bias[6]. We observe a slight asymmetry of the plotted estimates (Supplementary Figure 4), with a few outlying values that are large and positive, the most important among these being effect sizes from studies on refrigerators and air-conditioners (which have also been detected as outliers when we calculated Cook's distance).

We also use statistical tests more formally than the funnel plot to confirm the presence of publication bias and to estimate the 'true' average effect size adjusted for publication bias. We use PET (precision-effect test) and PEESE (precision-effect estimate with SE) for estimating/testing the 'true' effect in the presence of publication bias [7]. The coefficient of the standard error measures publication bias and can be thought of as a test of asymmetry in the funnel plot. As shown in Supplementary Table 12, the coefficient of standard error is indeed positive and significant, indicating presence of publication bias. The intercept of the models estimates the 'true' effect beyond publication bias. PET is a powerful test for the presence of an authentic effect beyond publication bias[8], but it tends to underestimate the true mean effect when there is a nonzero treatment effect. If an indication is given for the presence of a genuine effect, the intercept from the PEESE model is the preferred estimate[7]. As shown in Supplementary Table 12, the "true" mean effect estimated by this model is 0.09 which is slightly lower than the unadjusted mean effect of 0.10 though the confidence intervals overlap significantly. Therefore, while there is presence of publication, adjusting for it does not alter the main results. This remains true even when the intervention-specific results are adjusted for possible publication bias indicating a robust finding.

Supplementary Figure 4: Funnel plots with standard error of each effect size on the y-axis and z-value of the effect size on the x-axis.

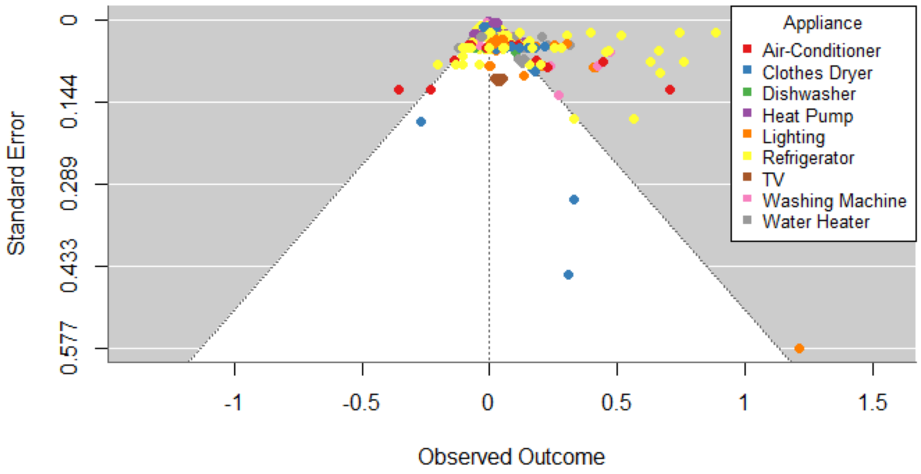

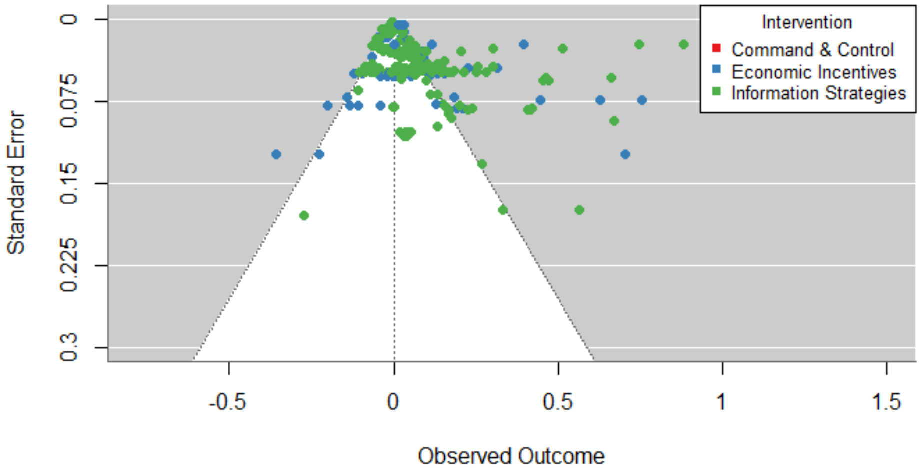

Supplementary Table 12: Average effect size across all interventions before and after correcting for publication bias

	estimate	se	z-value	p-value	ci.lb	ci.ub	
Base Model: Average treatment effect							
Mean estimate	0.10	0.02	5.98	<.01	0.07	0.14	***
Model PET: Average treatment effect with Standard Error as moderator							
Mean estimate	0.02	0.02	1.04	0.3	-0.02	0.07	

Standard Error	1.64	0.37	4.38	<.01	0.9	2.37	***
Model PEESE: Average treatment effect with Square of Standard Error as moderator							
Mean estimate ("True Effect")	0.09	0.02	5.38	<.01	0.06	0.12	***
Square of Standard Error	3.44	1.26	2.74	<.01	0.98	5.9	*

Supplementary Note 3: Machine learning and stopping criteria for abstract screening

We use an 'active-learning' algorithm to optimize the process of abstract screening, whereby a suite of traditional text classification models is trained at every iteration (11 SVM-based and tree-based models), using previously created labels as training data, and the best performing model is chosen for prediction of the next most likely relevant documents. We do not record performance metrics throughout every loop of active learning but provide a sample performance overview below.

Support Vector Classifiers (SVC) performs best overall, with the highest precision and a high recall, hence this model is used for prioritization. On average, approximately 14% to 23% of the items labeled as relevant by these models are relevant, depending on the model type. The SVC models correctly identify 46-75% of all relevant documents. These metrics provide a helpful overview but the errors in precision and recall are not directly propagated into the screening process as we still review a large number of abstracts manually and iteratively.

After training the model with 32 previously known relevant study abstracts and a random sample of 500 study abstracts, several reiterations were performed, where a team of five reviewers screened the predicted most relevant abstracts manually. The abstracts were screened individually, however, to ensure consistency, two samples of 50 studies were screened by the five reviewers independently at the abstract level (Kappa = 0.68).

We are sampling documents for screening from the entire pool of abstracts (N = 39,737). We use a formal approach in this review to stop screening at the point when the probability of finding more relevant studies at a given recall level is minimal. This point is determined using a statistical stopping criterion that ensures a recall of 95% achieved at p = 0.00 (Callaghan and Müller-Hansen 2020). In total, 5,861 studies were screened manually at the abstract level, with 614 rated as relevant. (Supplementary Figure 2). This resulted in around 85% work savings.

Supplementary References

1. Khanna, T. M. *et al.* A multi-country meta-analysis on the role of behavioural change in reducing energy consumption and CO2 emissions in residential buildings. *Nat. Energy* **6**, 925–932 (2021).
2. Delmas, M. A., Fischlein, M. & Asensio, O. I. Information strategies and energy conservation behavior: A meta-analysis of experimental studies from 1975 to 2012. *Energy Policy* **61**, 729–739 (2013).
3. Buckley, P. Prices, information and nudges for residential electricity conservation: A meta-analysis. *Ecol. Econ.* **172**, 106635 (2020).
4. Lohmann, P. M., Pizzo, A., Bauer, J. M., Khanna, T. M. & Reisch, L. A. Demand-side interventions for sustainable food systems: A meta-analysis of food-policy interventions targeting food consumption and waste behaviours. SSRN Scholarly Paper at https://doi.org/10.2139/ssrn.4811931 (2024).
5. Javaid, A., Khanna, T., Franza, M. & Creutzig, F. Behavioural interventions change individual transport choices but have a limited impact on transport mode split. Evidence from a systematic review. Preprint at https://doi.org/10.21203/rs.3.rs-2084989/v1 (2022).
6. Egger, M., Smith, G. D., Schneider, M. & Minder, C. Bias in meta-analysis detected by a simple, graphical test. *BMJ* **315**, 629–634 (1997).
7. Stanley, T. D. & Doucouliagos, H. Meta-regression approximations to reduce publication selection bias. *Res. Synth. Methods* **5**, 60–78 (2014).
8. Stanley, T. D. Meta-Regression Methods for Detecting and Estimating Empirical Effects in the Presence of Publication Selection*. *Oxf. Bull. Econ. Stat.* **70**, 103–127 (2008).

References to included papers

Usage:

1. A. Alberini, W. Gans, C. Towe, Free Riding, Upsizing, and Energy Efficiency Incentives in Maryland Homes. *The Energy Journal* **37**, 259–290 (2016).

2. A. Alberini, C. Towe, Information v. energy efficiency incentives: Evidence from residential electricity consumption in Maryland. *Energy Economics* **52**, S30–S40 (2015).

3. Z. Cheng, X. Zhang, J. Cai, Appliance energy efficiency policies and electricity consumption: Evidence from China. *Energy* **322**, 135740 (2025).

4. Y. Chuang, M. A. Delmas, S. Pincetl, Are Residential Energy Efficiency Upgrades Effective? An Empirical Analysis in Southern California. *Journal of the Association of Environmental and Resource Economists* **9**, 641–679 (2022).

5. N. Chun, Y. Jiang, How households in Pakistan take on energy efficient lighting technology. *Energy Economics* **40**, 277–284 (2013).

6. L. W. Davis, A. Fuchs, P. Gertler, Cash for Coolers: Evaluating a Large-Scale Appliance Replacement Program in Mexico. *American Economic Journal: Economic Policy* **6**, 207–238 (2014).

7. M. Hammerle, P. J. Burke, From natural gas to electric appliances: Energy use and emissions implications in Australian homes. *Energy Economics* **110**, 106050 (2022).

8. T. M. Khanna, *et al.*, Behavioral, Information, and Monetary Interventions to Reduce Energy Consumption in Households: A Living Systematic Review and Network Meta-Analysis. *Campbell Systematic Reviews* **21**, e70070 (2025).

9. B. Liddle, T. S. A. Loi, A. D. Owen, J. Tao, Evaluating consumption and cost savings from new air-conditioner purchases: The case of Singapore. *Energy Policy* **145**, 111722 (2020).

10. K. Mizobuchi, K. Takeuchi, Replacement or additional purchase: The impact of energy-efficient appliances on household electricity saving under public pressures. *Energy Policy* **93**, 137–148 (2016).

11. D. Naeher, R. Narayanan, V. Ziulu, Cash for Coolers or Sustainable Lighting? Assessing Different Components of a Large-Scale Energy Efficiency Program in Mexico. *The Journal of Development Studies* **60**, 479–493 (2024).

12. R. S. Ridge, Energy Conservation for Low-Income Households: The Evaporative Cooler Experience. *Eval Rev* **12**, 170–185 (1988).

13. E. Shojaeddini, B. Gilbert, Heterogeneity in the Rebound Effect: Evidence from Efficient Lighting Subsidies. *Environ Resource Econ* **84**, 173–217 (2023).

14. B. Sun, Heterogeneous direct rebound effect: Theory and evidence from the Energy Star program. *Energy Economics* **69**, 335–349 (2018).

15. X.-L. Yao, Y. Liu, X. Yan, A quantile approach to assess the effectiveness of the subsidy policy for energy-efficient home appliances: Evidence from Rizhao, China. *Energy Policy* **73**, 512–518 (2014).

Market share:

1. T. Buettner, B. Madzharova, Promoting Sales of Energy Efficient Household Appliances: Outcomes and Cost-Effectiveness of Rebate Programs.

2. S. Datta, M. Filippini, Analysing the impact of ENERGY STAR rebate policies in the US. *Energy Efficiency* **9**, 677–698 (2016).

3. S. Datta, S. Gulati, Utility rebates for ENERGY STAR appliances: Are they effective? *Journal of Environmental Economics and Management* **68**, 480–506 (2014).

4. S. Houde, J. E. Aldy, Consumers' Response to State Energy Efficient Appliance Rebate Programs. *American Economic Journal: Economic Policy* **9**, 227–255 (2017).

5. J. Schleich, A. Durand, H. Brugger, How effective are EU minimum energy performance standards and energy labels for cold appliances? *Energy Policy* **149**, 112069 (2021).

6. B. Wang, N. Deng, X. Liu, Q. Sun, Z. Wang, Effect of energy efficiency labels on household appliance choice in China: Sustainable consumption or irrational intertemporal choice? *Resources, Conservation and Recycling* **169**, 105458 (2021).

Purchase:

1. A. Alberini, A. Bigano, M. Boeri, Looking for free riding: energy efficiency incentives and Italian homeowners. *Energy Efficiency* **7**, 571–590 (2014).

2. H. Allcott, R. L. Sweeney, The Role of Sales Agents in Information Disclosure: Evidence from a Field Experiment. *Management Science* **63**, 21–39 (2017).

3. H. Allcott, D. Taubinsky, Evaluating Behaviorally Motivated Policy: Experimental Evidence from the Lightbulb Market. *American Economic Review* **105**, 2501–2538 (2015).

4. C. D. Anderson, J. D. Claxton, Barriers to Consumer Choice of Energy Efficient Products. *J CONSUM RES* **9**, 163 (1982).

5. M. A. Andor, A. Gerster, L. Götte, How effective is the European Union energy label? Evidence from a real-stakes experiment. *Environ. Res. Lett.* **14**, 044001 (2019).

6. M. Asinyaka, Willingness to Pay for Energy Efficient Refrigerating Appliances in Accra, Ghana: A Choice Experiment Approach. *Review of Economics* **70**, 15–39 (2019).

7. K. Caldwell, L. Vaughn, E. Harrod, J. Harrod, Social Marketing–Enhanced Home Energy Education Encourages Adoption of Energy-Saving Practices. *JOE* **57** (2019).

8. S. Ceolotto, E. Denny, Putting a New 'Spin' on Energy Information: Measuring the Impact of Reframing Energy Efficiency Information on Tumble Dryer Choices in a Multi-country Experiment. *J Consum Policy* **47**, 51–108 (2024).

9. L. W. Davis, G. E. Metcalf, NBER WORKING PAPER SERIES.

10. M. Del Mar Solà, A. De Ayala, I. Galarraga, The Effect of Providing Monetary Information on Energy Savings for Household Appliances: A Field Trial in Spain. *J Consum Policy* **44**, 279–310 (2021).

11. E. Denny, Long-term Energy Cost Labelling for Appliances: Evidence from a Randomised Controlled Trial in Ireland. *J Consum Policy* **45**, 369–409 (2022).

12. M. Deutsch, The effect of life-cycle cost disclosure on consumer behavior: evidence from a field experiment with cooling appliances. *Energy Efficiency* **3**, 303–315 (2010).

13. T. Dieu-Hang, R. Q. Grafton, R. Martínez-Espiñeira, M. Garcia-Valiñas, Household adoption of energy and water-efficient appliances: An analysis of attitudes, labelling and complementary green behaviours in selected OECD countries. *Journal of Environmental Management* **197**, 140–150 (2017).

14. C. Faure, M.-C. Guetlein, J. Schleich, Effects of rescaling the EU energy label on household preferences for top-rated appliances. *Energy Policy* **156**, 112439 (2021).

15. A. Figueroa, L. De Molière, A. Pegels, B. Never, F. Kutzner, Show me (more than) the money! Assessing the social and psychological dimensions to energy efficient lighting in Kenya. *Energy Research & Social Science* **47**, 224–232 (2019).

16. I. Galarraga, D. R. Heres, M. Gonzalez-Eguino, Price premium for high-efficiency refrigerators and calculation of price-elasticities for close-substitutes: a methodology using hedonic pricing and demand systems. *Journal of Cleaner Production* **19**, 2075–2081 (2011).

17. I. Galarraga, M. González-Eguino, A. Markandya, Willingness to pay and price elasticities of demand for energy-efficient appliances: Combining the hedonic approach and demand systems. *Energy Economics* **33**, S66–S74 (2011).

18. Y. Gao, M. Tavoni, Forget-Me-Not: The Persistent Effect of Information Provision for Adopting Climate-Friendly Goods. *Management Science* **70**, 4480–4501 (2024).

19. H. Goto, M. Goto, T. Sueyoshi, Consumer choice on ecologically efficient water heaters: Marketing strategy and policy implications in Japan. *Energy Economics* **33**, 195–208 (2011).

20. B. R. Hafner, D. Elmes, D. Read, M. P. White, Exploring the role of normative, financial and environmental information in promoting uptake of energy efficient technologies. *Journal of Environmental Psychology* **63**, 26–35 (2019).

21. H. Harajli, A. Chalak, Willingness to Pay for Energy Efficient Appliances: The Case of Lebanese Consumers. *Sustainability* **11**, 5572 (2019).

22. S. L. Heinzle, R. Wüstenhagen, Dynamic Adjustment of Eco-labeling Schemes and Consumer Choice – the Revision of the EU Energy Label as a Missed Opportunity? *Bus Strat Env* **21**, 60–70 (2012).

23. M. Jain, A. B. Rao, A. Patwardhan, Consumer preference for labels in the purchase decisions of air conditioners in India. *Energy for Sustainable Development* **42**, 24–31 (2018).

24. G. Jeong, Y. Kim, The effects of energy efficiency and environmental labels on appliance choice in South Korea. *Energy Efficiency* **8**, 559–576 (2015).

25. S. Kallbekken, H. Sælen, E. A. T. Hermansen, Bridging the Energy Efficiency Gap: A Field Experiment on Lifetime Energy Costs and Household Appliances. *J Consum Policy* **36**, 1–16 (2013).

26. S. Kuhn, J. Thøgersen, F. Kutzner, No trust in the choice architect? No problem! On the minor role of trust for the effectiveness of default interventions promoting the choice of energy-efficient appliances. *Journal of Environmental Psychology* **91**, 102115 (2023).

27. X. Li, C. D. Clark, K. L. Jensen, S. T. Yen, The Effect of Mail-in Utility Rebates on Willingness-to-Pay for ENERGY STAR $$^{\textregistered }$$ ® Certified Refrigerators. *Environ Resource Econ* **63**, 1–23 (2016).

28. Z. Li, X. Cao, Effectiveness of China's Labeling and Incentive Programs for Household Energy Conservation and Policy Implications. *Sustainability* **13**, 1923 (2021).

29. X. Liu, Z. Jin, Visualisation approach and economic incentives toward low carbon practices in households: A survey study in Hyogo, Japan. *Journal of Cleaner Production* **220**, 298–312 (2019).

30. B. Ma, Y. Yu, F. Urban, Green transition of energy systems in rural China: National survey evidence of households' discrete choices on water heaters. *Energy Policy* **113**, 559–570 (2018).

31. A. Mekonnen, S. Hassen, M. Jaime, M. Toman, X.-B. Zhang, The effect of information and subsidy on adoption of solar lanterns: An application of the BDM bidding mechanism in rural Ethiopia. *Energy Economics* **124**, 106869 (2023).

32. R. Nakano, *et al.*, Determinants of energy savings in Indonesia: The case of LED lighting in Bogor. *Sustainable Cities and Society* **42**, 184–193 (2018).

33. C. Neves, T. Oliveira, Drivers of consumers' change to an energy-efficient heating appliance (EEHA) in households: Evidence from five European countries. *Applied Energy* **298**, 117165 (2021).

34. R. G. Newell, J. Siikamäki, Nudging Energy Efficiency Behavior: The Role of Information Labels. *Journal of the Association of Environmental and Resource Economists* **1**, 555–598 (2014).

35. D. Nishijima, S. Kagawa, K. Nansai, M. Oguchi, Effects of product replacement programs on climate change. *Journal of Cleaner Production* **221**, 157–166 (2019).

36. C. Orset, Is information a good policy instrument to influence the energy behaviour of households? *Energy Economics* **102**, 105451 (2021).

37. M. A. S. Razali, M. Kamaludin, A. A. Azlina, Consumer Preference for Energy Label in the Purchase Decision of Refrigerator: A Discrete Choice Experiment Approach in the East Coast, Malaysia. *IJEEP* **12**, 441–450 (2022).

38. D. Revelt, K. Train, Mixed Logit with Repeated Choices: Households' Choices of Appliance Efficiency Level. *Review of Economics and Statistics* **80**, 647–657 (1998).

39. K. Sammer, R. Wüstenhagen, The influence of eco-labelling on consumer behaviour – results of a discrete choice analysis for washing machines. *Bus Strat Env* **15**, 185–199 (2006).

40. P. W. Schultz, *et al.*, Using Social Marketing to Spur Residential Adoption of ENERGY STAR® -Certified LED Lighting. *Social Marketing Quarterly* **21**, 61–78 (2015).

41. J. Shen, T. Saijo, Does an energy efficiency label alter consumers' purchasing decisions? A latent class approach based on a stated choice experiment in Shanghai. *Journal of Environmental Management* **90**, 3561–3573 (2009).

42. X. Shen, Y. L. Qiu, P. Liu, A. Patwardhan, The Effect of Rebate and Loan Incentives on Residential Heat Pump Adoption: Evidence from North Carolina. *Environ Resource Econ* **82**, 741–789 (2022).

43. M. Skourtos, D. Damigos, C. Tourkolias, A. Kontogianni, Efficient energy labelling: the impact of information content and style on product choice. *Energy Efficiency* **14**, 58 (2021).

44. M. D. M. Solà, M. Escapa, I. Galarraga, Effectiveness of monetary information in promoting the purchase of energy-efficient appliances: Evidence from a field experiment in Spain. *Energy Research & Social Science* **95**, 102887 (2023).

45. M. Stadelmann, R. Schubert, How Do Different Designs of Energy Labels Influence Purchases of Household Appliances? A Field Study in Switzerland. *Ecological Economics* **144**, 112–123 (2018).

46. K. E. Train, T. Atherton, Rebates, Loans, and Customers' Choice of Appliance Efficiency Level: Combining Stated-and Revealed-Preference Data. *The Energy Journal* **16**, 55–69 (1995).

47. J. Wang, Impact of incentives to purchase energy-efficient products: evidence from Chinese households based on a mixed logit model. *Energy Efficiency* **16**, 81 (2023).

48. D. O. Ward, C. D. Clark, K. L. Jensen, S. T. Yen, C. S. Russell, Factors influencing willingness-to-pay for the ENERGY STAR ® label. *Energy Policy* **39**, 1450–1458 (2011).

49. N. Wasi, R. T. Carson, The influence of rebate programs on the demand for water heaters: The case of New South Wales. *Energy Economics* **40**, 645–656 (2013).

50. D. Zha, G. Yang, W. Wang, Q. Wang, D. Zhou, Appliance energy labels and consumer heterogeneity: A latent class approach based on a discrete choice experiment in China. *Energy Economics* **90**, 104839 (2020).

51. H. Zhou, J. O. Bukenya, Information inefficiency and willingness-to-pay for energy-efficient technology: A stated preference approach for China Energy Label. *Energy Policy* **91**, 12–21 (2016).
